# Upwelling, climate change, and the shifting geography of coral reef development

**DOI:** 10.1038/s41598-023-28489-0

**Published:** 2023-02-07

**Authors:** Victor Rodriguez-Ruano, Lauren T. Toth, Ian C. Enochs, Carly J. Randall, Richard B. Aronson

**Affiliations:** 1 Department of Ocean Engineering and Marine Sciences, Florida Institute of Technology, 150 West University Boulevard, Melbourne, FL 32901 USA; 2 U.S. Geological Survey, St. Petersburg Coastal and Marine Science Center, 600 4th St. South, St. Petersburg, FL 33701 USA; 3 3NOAA, Atlantic Oceanographic and Meteorological Laboratory, Ocean Chemistry and Ecosystem Division, 4301 Rickenbacker Cswy., Miami, FL 33149 USA; 4 Australian Institute of Marine Science, PMB No. 3, Townsville, QLD 4810 Australia

**Keywords:** Ecology, Climate-change ecology, Palaeoecology, Tropical ecology, Geology

## Abstract

The eastern tropical Pacific is oceanographically unfavorable for coral-reef development. Nevertheless, reefs have persisted there for the last 7000 years. Rates of vertical accretion during the Holocene have been similar in the strong-upwelling Gulf of Panamá (GoP) and the adjacent, weak-upwelling Gulf of Chiriquí (GoC); however, seasonal upwelling in the GoP exacerbated a climate-driven hiatus in reef development in the late Holocene. The situation is now reversed and seasonal upwelling in the GoP currently buffers thermal stress, creating a refuge for coral growth. We developed carbonate budget models to project the capacity of reefs in both gulfs to keep up with future sea-level rise. On average, the GoP had significantly higher net carbonate production rates than the GoC. With an estimated contemporary reef-accretion potential (RAP) of 5.5 mm year^−1^, reefs in the GoP are projected to be able to keep up with sea-level rise if CO_2_ emissions are reduced, but not under current emissions trajectories. With an estimated RAP of just 0.3 mm year^−1^, reefs in the GoC are likely already unable to keep up with contemporary sea-level rise in Panamá (1.4 mm year^−1^). Whereas the GoP has the potential to support functional reefs in the near-term, our study indicates that their long-term persistence will depend on reduction of greenhouse gases.

## Introduction

Coral reefs provide key ecosystem services to coastal communities, including fisheries, tourism, and protection from storms^1,2^. These services rely on the ability of stony corals to accumulate calcium-carbonate (CaCO_3_) skeletons through time and produce a complex, three-dimensional framework^3,4^. The accumulation of reef framework is reduced by the destructive processes of erosion by biological and physical agents, and abiotic dissolution^5–7^. Bioerosion is one of the most important and persistent drivers of reef-framework removal^5^. A myriad of organisms contribute to this process, including fish and invertebrate grazers that scrape the reef substrate, invertebrates that bore into coral skeletons for refuge, and microbes that colonize and dissolve dead coral skeletons^8^.

When calcification exceeds erosion, a coral reef exhibits net accretion. When rates of erosion exceed rates of calcification, however, net erosion drives a loss of reef-framework habitat over time^9^. The capacity of reefs to break waves and protect coastlines is contingent on their ability to keep up with sea-level rise through net accretion^2^. Identifying which reefs will be able to grow fast enough to keep up with current and future sea-level rise^10^ and which reefs will likely drown is, therefore, essential for identifying the regions and human populations most vulnerable to climate change.

Most reefs in the Caribbean region are currently in a net-erosional or net-neutral state, which is a product of decreasing coral cover from coral bleaching and disease outbreaks^11,12^. Similarly, some reefs in the central Pacific are threatened by increasing thermal stress and predator outbreaks, and they are likewise experiencing low carbonate-production rates^13,14^. On the other hand, reefs in the western Pacific have experienced fewer thermal-stress events and disease outbreaks than the central Pacific and the Caribbean; these reefs generally maintain high rates of carbonate production^14,15^.

Disturbances that drive coral mortality, such as the thermal-stress events that cause coral bleaching, can shift a reef’s carbonate budget from a net-positive (i.e., accretionary) to a net-negative (i.e., erosional) state^16,17^. Once such a disturbance ceases, coral populations can potentially recover to pre-disturbance conditions^18^; however, global climate change is promoting an increase in the frequency and severity of thermal-stress events, and many coral populations no longer have sufficient time to recover between disturbances^12,19^.

Coral reefs of the eastern tropical Pacific (ETP) are exposed to highly variable environmental conditions that are marginal for coral growth^20,21^. The ETP is subject to varying degrees of seasonal upwelling, thermal anomalies from El Niño–Southern Oscillation (ENSO) events, and large tidal ranges. These oceanographic phenomena can be accompanied by large fluctuations in temperature, salinity, aragonite saturation state (Ω_aragonite_), nutrients, and turbidity, all of which are stressful for corals^22–25^. Because of the regionally high turbidity, coral reefs in the ETP are largely constrained to a relatively narrow depth range, with most reef development occurring within 10 m of sea level^20,26,27^. Additionally, the narrow continental shelf of the ETP drops abruptly to the deep seafloor, restricting the area that is available for reef development^21^. As a result of all these factors, reefs in the ETP may be particularly vulnerable to sea-level rise. Nevertheless, reef development in the region has been possible, albeit intermittently, for the last 7000 years^27,28^.

Pocilloporids generally dominate the shallow habitats of reefs in the ETP (0–5 m), including the reef crest and upper forereef slope, whereas deeper habitats (5–15 m) are usually dominated by massive coral taxa, including *Porites* spp., *Pavona* spp., and *Gardineroseris planulata*^26^. Although some reefs in the ETP are dominated by massive corals^29^, the known Holocene frameworks of shallow reefs in Pacific Panamá are composed of uncemented, interlocking, branching skeletons of *Pocillopora* spp. packed in a sandy-mud to muddy-sand matrix^30^.

The major environmental constraints on reef development in the ETP have produced a patchy distribution of pocilloporid reefs across the region^21,27^. As a result of a low regional Ω_aragonite_, submarine cementation is relatively low^24^. Seasonal upwelling events in the ETP further hinder coral growth via cold-water stress, decreased Ω_aragonite_, and high nutrient concentrations^24,31–33^. In addition, high nutrient concentrations driven by upwelling promote higher abundances of heterotrophic, macroboring invertebrates, promoting higher rates of bioerosion than in areas that experience weak to no upwelling^7^. Indeed, Enochs et al.^7^ reported significantly higher macroboring rates in ETP reefs that experience stronger upwelling. These results are in agreement with data from the Great Barrier Reef, where there was a higher infestation of carbonate substrate by macroborers in inshore, nutrient-rich reefs than in offshore, nutrient-poor reefs^34^.

**Figure 1:**
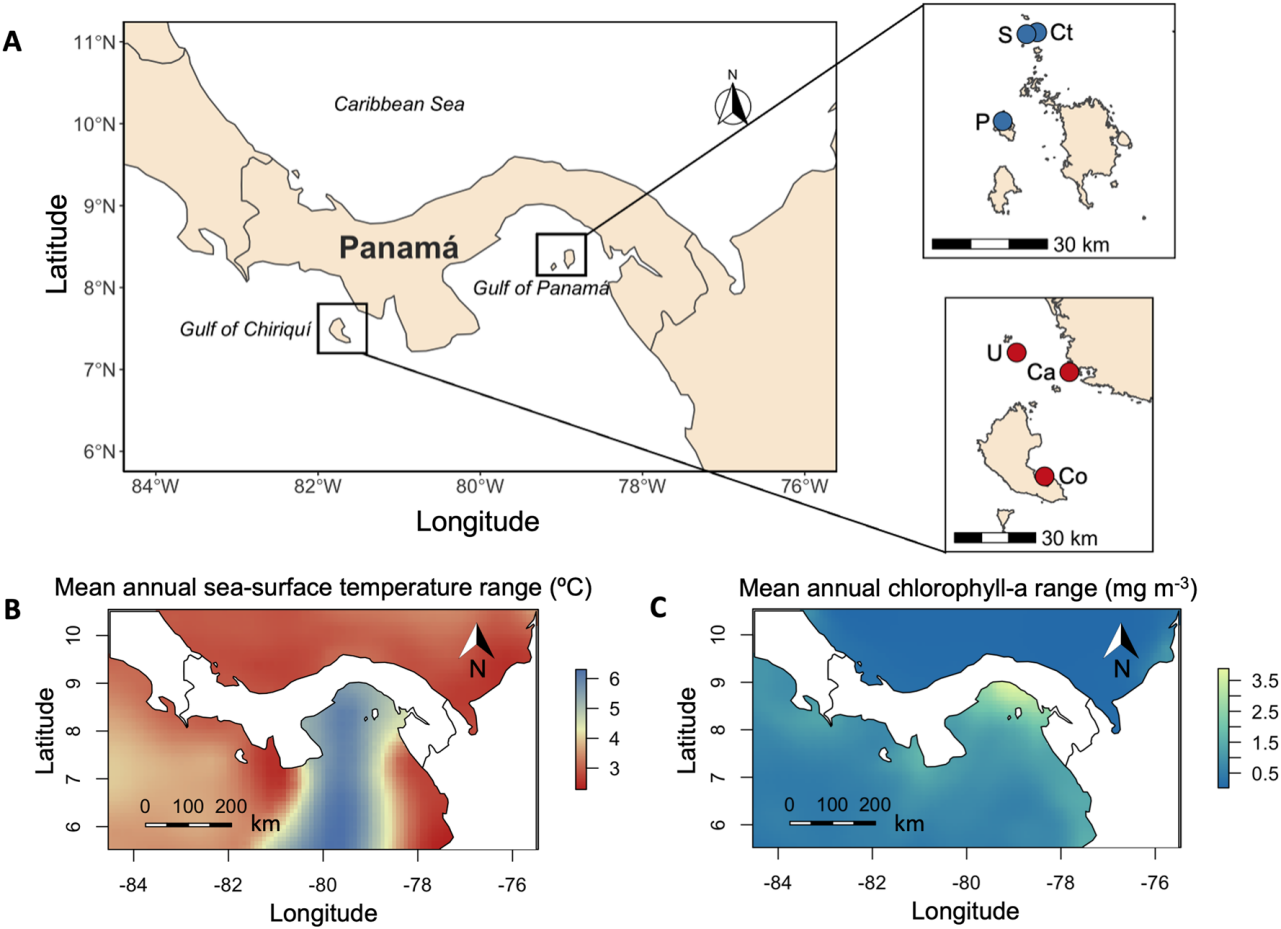
(**A**) Map of Pacific Panamá; (**B**) the average annual range in sea-surface temperature values (average for 2000–2014, in °C); (**C**) the average annual range in chlorophyll-*a* values (average for 2000–2014, in mg m^−3^). In (**A**), the study sites within each gulf are shown as insets. Red circles represent the sites in the Gulf of Chiriquí and blue circles represent the sites in the Gulf of Panamá. In the Gulf of Chiriquí: Co = Coiba, Ca = Canales de Tierra, U = Uva. In the Gulf of Panamá: P = Pedro González, S = Saboga, Ct = Contadora. Data for panels (**B**) and (**C**) were retrieved from the Bio-ORACLE Marine Database^37,38^. These maps were generated in R version 4.2.2 (Ref.^39^) using the “ggplot2” (https://ggplot2.tidyverse.org, https://github.com/tidyverse/ggplot2; Ref.^40^) and “ggspatial” (https://paleolimbot.github.io/ggspatial/; Ref.^41^) packages.

Pacific Panamá consists of two major gulfs with different oceanographic conditions: the Gulf of Panamá (GoP) and the Gulf of Chiriquí (GoC; Fig. 1A). The GoP is subject to strong, seasonal, wind-driven upwelling, which produces broad ranges of sea-surface temperature (SST range 21–29 °C; Fig. 1B), nutrients (leading to chlorophyll-*a* concentrations of 0.5–3.5 mg m^−3^; Fig. 1C), and carbonate chemistry (Ω_aragonite_ range 2.96–2.79; Refs.^7,24^). In contrast, the GoC is a weak-upwelling system that maintains warm SSTs, and relatively constant nutrients and carbonate chemistry year-round^24,35,36^. Holocene paleoecological records indicate that reefs in the GoP have significantly less framework accumulation than the reefs in the GoC^27,30^.

Reefs throughout Pacific Panamá experienced a hiatus in reef development of ~ 2300 years beginning ~ 4100 years ago as a result of high ENSO variability^42^. Because of intense upwelling in the GoP, the hiatus began earlier and lasted longer there than in the GoC^42,43^. These patterns indicate that seasonal upwelling has been a major driver of reef development in the ETP during the Holocene^44^.

At present, however, upwelling in the GoP is buffering corals from warming associated with human-induced climate change^36^ (but see Ref.^45^). According to SST trends over the last 150 years, the GoC is warming at a faster rate than the GoP, and thermal conditions are now more favorable for coral survival and growth in the GoP than in the GoC^36,46^. During the El Niño event of 2015–2016, a significantly higher proportion of corals bleached in the GoC than in the GoP, and corals in the GoP grew faster than corals in the GoC two years after the event. Together, these results imply that seasonal upwelling in the GoP mitigated the thermal stress of the El Niño event^36^.

The variability in coral survival between the two gulfs provides insights into the persistence of coral reefs under different degrees of upwelling intensity. In this study, we aimed to determine whether there is currently a difference between the gulfs in reef-accretion potential (RAP; Ref.^12^), which is an estimate of the maximum vertical accretion a reef can achieve. The RAPs were compared with future rates of sea-level rise predicted for different representative concentration pathways (RCPs) by the Intergovernmental Panel on Climate Change (IPCC; Ref.^10^). RCPs predict future greenhouse-gas concentrations under different emissions scenarios. Comparing the RAPs of reefs in the two gulfs under these RCPs allows us to predict how upwelling will influence the ability of Panamá’s reefs to keep up with projected rates of sea-level rise under different climate-change scenarios.

In a previous study, the benthic assemblages of three shallow reef slopes were monitored within each gulf from spring 2016 to spring 2018 to evaluate the impacts of the 2015‒2016 El Niño event^36^, and we used the ecological surveys from that study to quantify calcification and erosion processes and construct carbonate budgets. In the GoP, we surveyed the reefs at Saboga, Contadora, and Pedro Gonzalez Islands in the Pearl Islands Archipelago. In the GoC, we surveyed the reefs at Uva, Coiba, and Canales de Tierra (Fig. 1). In addition to these surveys, we incorporated data from fish and sea-urchin surveys, and palaeoecological data from reef cores to account for most of the variables that influence reef accretion^7,42^. We also quantified in situ calcification rates for *Pocillopora* spp. (henceforth *Pocillopora*), which is the dominant coral taxon of these reefs, to account for local variability in growth potential. Because of the phenotypic plasticity that pocilloporids exhibit in the ETP^47^, it is challenging to identify species based solely on morphology. The calcification rates we provide are for the genus *Pocillopora*. Calcification rates among *Pocillopora* morphotypes in the ETP are similar enough to justify genus-level averaging^48–50^. We hypothesized that thermal stress would prevent corals in the GoC from growing rapidly enough to keep up with future rates of sea-level rise. Seasonal upwelling in the GoP, however, could serve as a refuge for coral survival and as a location where at least some coral reefs will maintain rates of carbonate production high enough to keep pace with projected sea-level rise.

## Results

### *Pocillopora* calcification estimates

**Figure 2:**
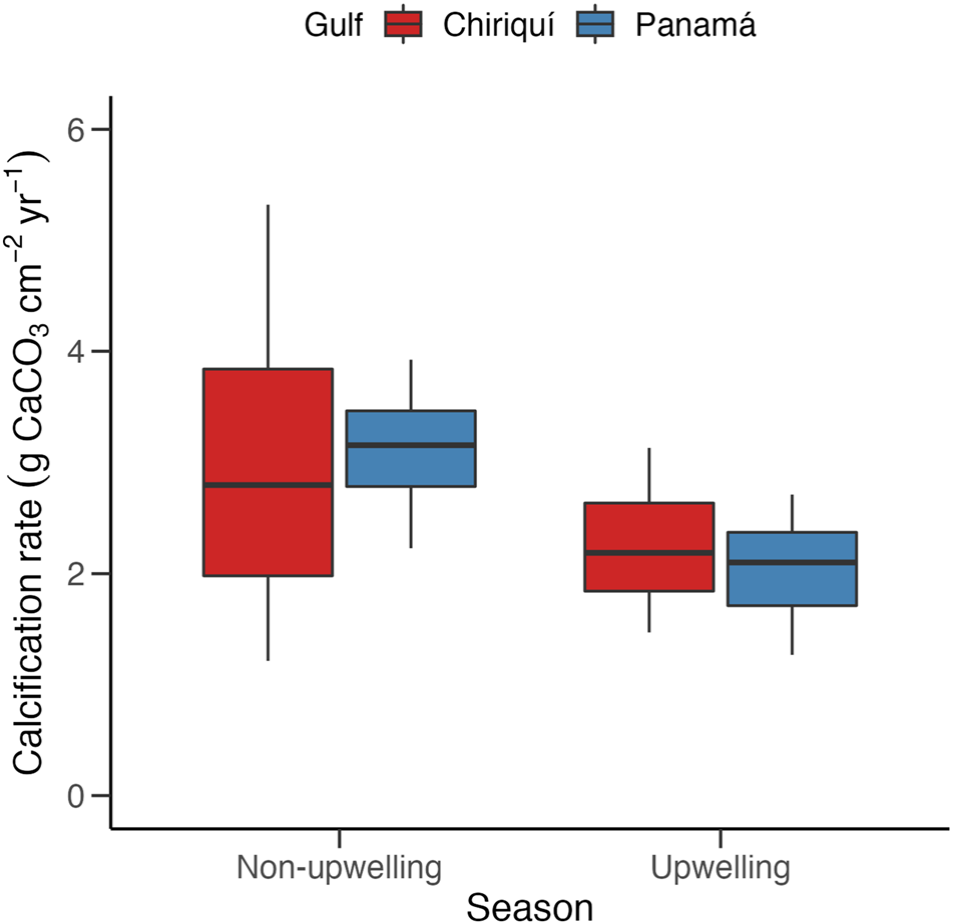
Boxplot of median (± interquartile range) *Pocillopora* calcification rate (g CaCO_3_ cm^−2^ year^−1^) for each gulf across the upwelling and non-upwelling seasons of Pacific Panamá from 2016 to 2018. The upwelling season runs from March through September, and the non-upwelling season runs from October through February.

Gulf was not a significant predictor of calcification by *Pocillopora* (fixed effect of gulf: estimate = − 0.03, SE = 0.22, t_1,4_ = − 0.15, p = 0.89; mean ± SE: GoP = 2.2 ± 0.1 g CaCO_3_ cm^−2^ year^−1^; GoC = 2.1 ± 0.2 g CaCO_3_ cm^−2^ year^−1^). Similarly, there was no significant difference in calcification of *Pocillopora* among years from 2016‒2018 (fixed effect of year: estimate = -0.38, SE = 0.22, t_1,41_ = -1.77, p = 0.08), but there was a significant effect of season (fixed effect of season: estimate = -0.68, SE = 0.22, t_1,22_ = -3.04, p < 0.01), with higher calcification rates in both gulfs during the non-upwelling season (spring–autumn; March to September; Fig. 2). The average calcification rate of *Pocillopora* in the GoP dropped by 35%, from 3.1 ± 0.1 g CaCO_3_ cm^−2^ year^−1^ in the non-upwelling season, to 2.0 ± 0.1 g CaCO_3_ cm^−2^ year^−1^ in the upwelling season (autumn–spring; September to March). Similarly, average calcification rates in the GoC dropped by 23%, from 2.9 ± 0.3 g CaCO_3_ cm^−2^ year^−1^ in the non-upwelling season, to 2.3 ± 0.03 g CaCO_3_ cm^−2^ year^−1^ in the upwelling season. Gulf was not a significant predictor of seasonal calcification (fixed effect of gulf: estimate = 0.2, SE = 0.19, t_1,64_ = 1.07, p = 0.29).

### Gross carbonate production

**Figure 3:**
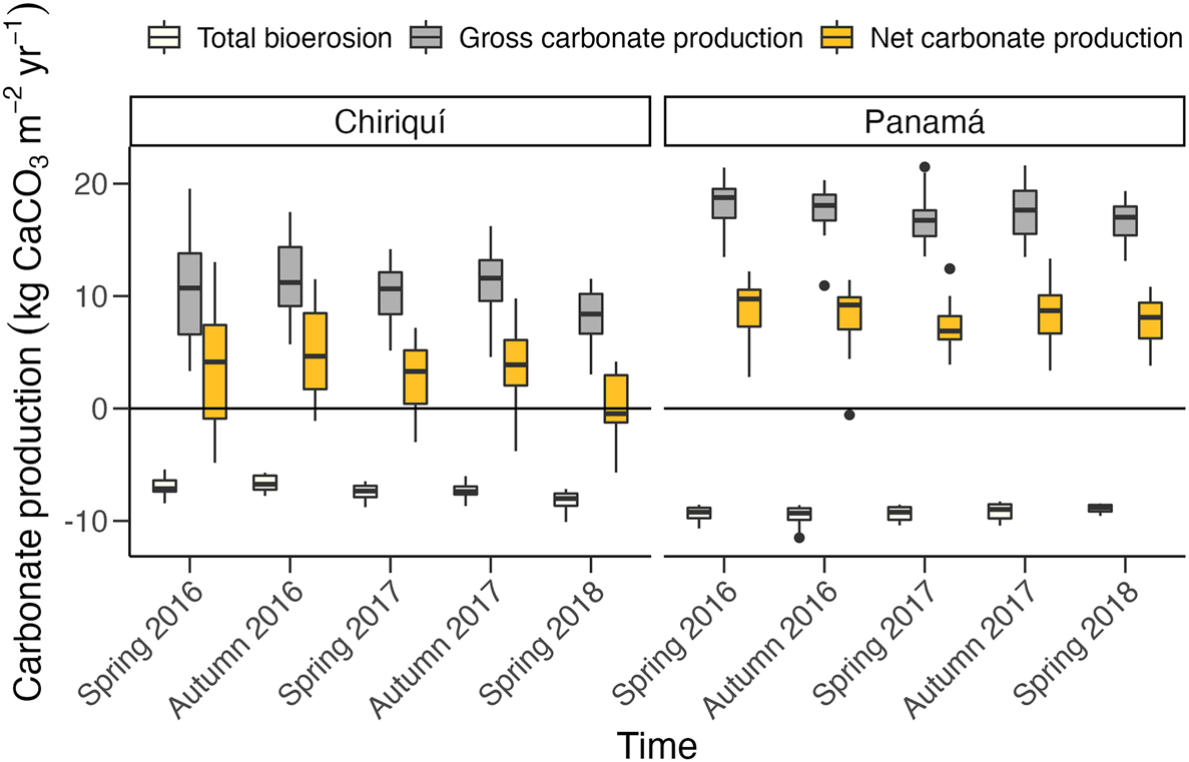
Boxplot of median (± interquartile range) gross carbonate production (gray boxes), total bioerosion (white boxes), and net carbonate production (yellow boxes) for each gulf across the five surveys from 2016–2018. All of the rates are reported in kg CaCO_3_ m^−2^ year^−1^. The black horizontal line delimits the division between net production and net erosion. Black points represent statistical outliers.

Gross carbonate production was significantly higher in the GoP than in the GoC (fixed effect of gulf: estimate = 6.97, SE = 1.22, t_1,4_ = 5.71, p < 0.01; Fig. 3). This result was driven by the higher average coral cover in the GoP (78%) than the GoC (50%; Ref.^36^). *Pocillopora* were responsible for > 90% of carbonate production in both gulfs. Gross carbonate production significantly declined through time in both gulfs (fixed effect of time: spring 2018 estimate = -1.93, SE = 0.64, t_4,165_ = − 3.03, p < 0.01), with an 8% decrease in the GoP (Spring 2016 = 18.2 ± 0.5 kg CaCO_3_ m^−2^ year^−1^; Spring 2018 = 16.7 kg CaCO_3_ m^−2^ year^−1^ ± 0.4 kg CaCO_3_ m^−2^ year^−1^) and a 24% decrease in the GoC (Spring 2016 = 10.7 ± 1.2 kg CaCO_3_ m^−2^ year^−1^; Spring 2018 = 8.1 ± 0.6 kg CaCO_3_ m^−2^ year^−1^; Fig. 3). These trends in gross carbonate production reflect the significant decrease in coral cover reported at these reefs by Randall et al.^36^. Both gulfs exhibited the highest deviation in gross carbonate production from the initial surveys during the spring of 2018 (fixed effect of time GoP; spring 2018 estimate = − 1.50, SE = 0.68, t_4,82_ = − 2.19, p < 0.05; fixed effect of time GoC: spring 2018 estimate = − 2.39, SE = 1.08, t_4,79_ = − 2.20, p < 0.05).

### Gross bioerosion

Bioerosion rates were significantly higher in the GoP than in the GoC (fixed effect of gulf: estimate = 1.92, SE = 0.20, t_1,4_ = 9.64, p < 0.001; Fig. 3). Bioerosion rates increased significantly through time in the GoC (fixed effect of time; spring 2018 estimate GoC = 1.15, SE = 0.25, t_4,79_ = 4.56, p < 0.0001), and decreased significantly through time in the GoP (fixed effect of time; spring 2018 estimate GoP = − 0.42, SE = 0.19, t_4,82_ = − 2.18, p < 0.05). The average bioerosion rate in the GoP decreased by 7% from − 9.6 ± 0.2 kg CaCO_3_ m^−2^ year^−1^ in the autumn of 2016 to − 8.9 ± 0.1 kg CaCO_3_ m^−2^ year^−1^ in the spring of 2018 (Fig. 3). Average bioerosion rates in the GoC increased by 17%, from − 6.6 ± 1.6 kg CaCO_3_ m^−2^ year^−1^ in the autumn of 2016 to − 8.1 ± 0.2 kg CaCO_3_ m^−2^ year^−1^ in the spring of 2018 (Fig. 3).

Macroborers were the dominant bioeroder group in both gulfs, contributing 88% of gross bioerosion in the GoP and 85% of gross bioerosion in the GoC across all time periods. Macroboring was significantly higher in the GoP than in the GoC (GoP = − 8.1 ± 0.1 kg CaCO_3_ m^−2^ year^−1^, GoC = − 6.3 ± 0.1 kg CaCO_3_ m^−2^ year^−1^; fixed effect of gulf: estimate = 26.66, SE = 2.83, t_1,4_ = 9.44, p < 0.001). The second-most-prominent source of bioerosion was dissolution by sponges, contributing 4% of gross bioerosion in the GoP and 5% of gross bioerosion in the GoC across all time periods. There was no significant difference in sponge dissolution rates between gulfs nor through time (fixed effect of time: Spring 2018 estimate = − 0.005, SE = 0.005, t_4,165_ = 1.08, p = 0.28; fixed effect of gulf: estimate = 0.006, SE = 0.009, t_1,4_ = 0.64, p = 0.56). Parrotfish were the third-most-prominent bioeroders, contributing 3% of gross bioerosion for both gulfs across all time periods. There was no significant difference in parrotfish bioerosion rates between gulfs (fixed effect of gulf: estimate = − 0.026, SE = 0.14, t_1,4_ = − 0.18, p = 0.07). Since the parrotfish assemblage was only surveyed during the spring of 2018 and extrapolated to the other time periods (assuming abundances remained stable through time; see Materials and Methods), we were not able to test for changes through time.

### Net carbonate production

Net carbonate-production rates were significantly higher in the GoP than in the GoC (fixed effect of gulf: estimate = 5.05, SE = 1.31, t_1,4_ = 3.85, p < 0.05; Fig. 3). There was also significant decline in net carbonate production through time (fixed effect of time; spring 2018 estimate = − 2.27, SE = 0.76, t_4,165_ = − 3.00, p < 0.01), driven by a 100% decrease in the GoC (fixed effect of time; spring 2018 estimate = − 3.54, SE = 1.26, t_4,79_ = − 2.81, p < 0.01). Average net carbonate production in the GoC declined from 4.7 ± 1.0 kg CaCO_3_ m^−2^ year^−1^ in the autumn of 2016 to 0.0 ± 0.7 kg CaCO_3_ m^−2^ year^−1^ in the spring of 2018. In the GoP, net carbonate-production declined by 11%, from 8.8 ± 0.6 kg CaCO_3_ m^−2^ year^−1^ in the spring of 2016 to 7.8 ± 0.5 kg CaCO_3_ m^−2^ year^−1^ in the spring of 2018.

### Threshold values and reef-accretion potential

Coral cover was a significant predictor of net carbonate production (effect of coral cover: estimate = 0.24, SE = 0.009, t_1,32_ = 25.17, p < 0.0001; Supplementary Table S1). We calculated that to maintain a net-positive carbonate budget, the reefs of the GoC would require coral cover of at least 40%, and the GoP would require 43%. Gulf and coral cover were both significant predictors of RAP (fixed effect of gulf: estimate = − 0.65, SE = 0.17, t_1,32_ = − 3.84, p < 0.001; fixed effect of coral cover: estimate = 0.16, SE = 0.004, t_1,32_ = 39.17, p < 0.0001; Supplementary Table S1). To keep up with local rates of sea-level rise projected by RCP 2.6 (3.3 mm year^−1^), we calculated that the GoC would require coral cover to be at least 59%, whereas the GoP would require coral cover to be 61%. To keep up with RCP 4.5 (5.7 mm year^−1^), the GoC would require coral cover to be at least 75%, and the GoP would require coral cover to be at least 76%. For RCP 8.5, estimated present day CaCO_3_ production is not sufficient for the reefs in Pacific Panamá to keep up with such a high rate of sea-level rise (12.3 mm year^−1^), even at 100% coral cover. We note that the threshold values for net carbonate production and RAP were calculated considering the contemporary status of the bioeroding assemblages; therefore, these models assume that bioeroding assemblages remain stable through time.

## Discussion

### Spatial and temporal trends in coral growth

Coral calcification in both gulfs was significantly lower during the upwelling season than during the non-upwelling season. This result was expected for the GoP, as previous studies have shown that cold water from strong, seasonal upwelling (December to mid-April), hinders coral growth there^31^. The seasonal decline in calcification in the GoC could have been due to several factors. Although upwelling is not as common in the GoC as in the GoP, the GoC does experience significant thermocline-shoaling in February and March^35^, so it is possible that the intrusion of cold, nutrient-rich and low-pH waters are also affecting coral calcification there. Additionally, due to logistical constraints, the autumn–spring ‘upwelling season’ in our study also incorporated the end of the non-upwelling ‘wet season’ (late-April to December) as well as the upwelling season. An increase in cloud cover during the wet season has been associated with regional decreases in coral growth in Pacific Panamá and Costa Rica^51,52^, which could have contributed to the seasonal decline in calcification in both gulfs.

Our calcification estimates based on buoyant weights and colony surface areas (GoP = 2.2 ± 0.1 g CaCO_3_ cm^−2^ year^−1^; GoC = 2.1 ± 0.2 g CaCO_3_ cm^−2^ year^−1^; mean ± SE) are consistent with a recent study that estimated calcification rates of 1.6–1.9 g CaCO_3_ cm^−2^ year^−1^ for pocilloporids in the Mexican ETP^53^. That study used a method that considers linear extension and the morphology of each species. Yet our rates are less than half of those previously estimated in Pacific Panamá using the product of skeletal extension rate (cm year^−1^) and bulk density (g cm^−3^), which reported calcification rates ranging from 5.2 to 6.0 g CaCO_3_ cm^−2^ year^−1^ for the 2003–2006 time period (Ref.^54^). The calcification rate in that study does not account for the influence of the three-dimensional morphology of branching taxa such as *Pocillopora*^54^. Furthermore, it relies on gross estimates of vertical extension and bulk skeletal density, which implicitly assumes that colonies are actively calcifying uniformly across their entire surface. Although this may be the case for massive corals, branching corals actively calcify at the branch tips but exhibit lower calcification rates across the remainder of the colony; therefore, the analysis likely overestimated calcification rates for branching species^55^. Our results are consistent with Manzello’s^54^ hypothesis predicting declines in coral calcification due to climate change^54^, but the different methods used among studies also likely contributed to the differences in calcification estimates. The buoyant-weight method used in our study is the only one that directly quantifies changes in aragonite mass through time (calcification) for corals^56,57^.

### Spatial and temporal trends in bioerosion

Rates of bioerosion in the ETP are much higher than the rates that have been estimated for other regions. Based on our most recent surveys, average bioerosion across both gulfs in Pacific Panamá is currently − 8.5 kg CaCO_3_ m^−2^ year^−1^ ± 0.1, which is more than double the bioerosion pressure estimated for the Caribbean (− 1.9 kg CaCO_3_ m^−2^ year^−1^), Indian Ocean (− 2.9 kg CaCO_3_ m^−2^ year^−1^), and west-central Pacific Ocean (− 1.5 kg CaCO_3_ m^−2^ year^−1^, Ref.^58^). Macroboring fauna accounted for 85–95% of the total bioerosion pressure across all reefs in our study. Total bioerosion, however, exhibited diverging trends in the two gulfs, with higher, but declining bioerosion in the GoP and lower, but increasing bioerosion in the GoC from 2016‒2018.

The higher bioerosion pressure in the GoP, especially from macroboring, is likely driven by high nutrient levels in the GoP during the upwelling season. Previous studies in Pacific Panamá and in other regions have shown that reefs subjected to high nutrient levels have higher abundances of suspension-feeding, macroboring fauna^7,59,60^. The decline in rates of bioerosion in the GoP was due to a decline in macroboring rates over time, likely driven by the decrease in cover of thick algal turfs observed over the course of the study. Dead reef framework covered by thick algal turfs generally harbors a higher abundance of macroboring fauna and therefore experiences higher macroboring rates than live or bare reef framework^16,61^. The decline of thick algal turfs in the GoP coincides with a significant increase in population densities of *Diadema mexicanum* (from 0.5 ind m^−2^ in the GoP in spring of 2016 to 1.7 ind m^−2^ in spring of 2018; Supplementary Fig. S1), which is the species of herbivorous sea urchin that likely controlled the growth of algal turfs^62^. For the GoC, the increase in bioerosion rates through time is due to a larger decrease in coral cover than the GoP (51% ± 5.9 to 39% ± 3.0 and 81% ± 2.3 to 75% ± 2.0, respectively), coupled with a more rapid increase in *D. mexicanum* densities than the GoP (from 0.4 ind m^−2^ in the GoP in spring 2016 to 4.9 ind m^−2^ in spring of 2018; Supplementary Fig. S1). This is a similar, albeit less intense, scenario to what occurred on Uva Island reef after the 1982–1983 ENSO event, when thermal stress caused a mass bleaching event that killed ~ 75% of all corals^63^. Following that event, the dead coral skeletons were colonized by macroalgae, and the overabundance of this resource led to a drastic increase in sea-urchin densities^64^. That sea-urchin outbreak caused significant destruction of the reef framework for the next 16 years^65^.

### Decadal-scale changes in carbonate budgets

Our net carbonate production estimates are within the range of those reported in previous studies in the region. For Uva Island reef in the GoC, Eakin^16^ reported a net carbonate production rate of 0.6 kg CaCO_3_ m^−2^ year^−1^ for the fore-reef prior to the 1982–1983 El Niño event. The 1982–1983 El Niño event caused significant thermal stress, high coral mortality, and an increase in sea-urchin bioerosion (Supplementary Fig. S2), which lowered net carbonate production at Uva Island reef to 0.1 kg CaCO_3_ m^−2^ year^−1^ (Refs.^16,63,64^). Although our net carbonate production estimates for Uva Island reef average 4.2 kg CaCO_3_ m^−2^ year^−1^ across all surveys between 2016 and 2018, our most recent ones from 2018 averaged just 0.9 kg CaCO_3_ m^−2^ year^−1^, which closely resembles Eakin’s estimates^16^. These relatively low values were driven by a decrease in coral cover (56% to 43%) and high rates of bioerosion, particularly from infauna^16^ (see Supplementary Table S2; Supplementary Methods). The low accretion estimates from Eakin’s and our model highlight bioerosion as a major control on reef accretion in the ETP. Both models also indicate that macroboring is the dominant bioerosional process (Supplementary Table S2). Although bioeroder assemblages can be highly variable in the short term—for example, in outbreaks of grazer populations following mass-coral-mortality events (Supplementary Fig. S2; Refs.^65,66^)—macroboring appears to be the dominant bioerosion pressure in the long term. The main difference between Eakin’s and our net rates of carbonate production stems from the methods used to estimate coral calcification. Whereas we estimated a calcification rate of 20.8 kg CaCO_3_ m^−2^ year^−1^ for *Pocillopora* using buoyant weight and surface area, Eakin estimated a calcification rate of 5.5 kg CaCO_3_ m^−2^ year^−1^ using the relationship between linear extension and skeletal density (Ref.^16^).

Reefs of the ETP have undergone decadal-scale episodes of decline and recovery as a consequence of ENSO-driven bleaching events^64,67–69^. At Uva Island reef, for example, the 1982–1983 El Niño event caused extensive coral bleaching, which killed 50% of the corals^63^. The following year, there was a major recruitment event of the sea urchin *D. mexicanum*, which led to severe bioerosion of the reef framework^64^. A gradual recovery in coral cover began in the early 1990s and the reef was largely unaffected by the 1997–1998 ENSO event^65^. Subsequent episodes of coral mortality occurred, but coral cover attained pre-disturbance values during the early 2010s^70^. Even though recovery trajectories have been slow for Uva Island reef, the reef has been relatively resilient to acute disturbance events thus far. Yet the current decline in carbonate production suggests that coral reefs in the GoC are vulnerable to future sea-level rise. A 10% decrease in coral cover between 2016 and 2018, coupled with a significant increase in sea-urchin abundance (Supplementary Fig. S1), caused carbonate productions of reefs in the GoC to shift from a net-positive state to a net-neutral state (Fig. 3). The reef at Canales de Tierra is already experiencing net erosion with an average net carbonate production of − 1.4 ± 1.5 kg CaCO_3_ m^−2^ year^−1^ for the spring of 2018 (Supplementary Table S3).

Although it took Uva Island reef several decades to recover to pre-disturbance levels of coral cover after the 1982–1983 event, the reef at Saboga in the GoP recovered from < 5% coral cover to pre-disturbance levels of 50% coral cover within 10 years^67^. Coral cover there has now increased to 75% and the average net carbonate-production rate during our most recent survey was 5.6 kg CaCO_3_ m^−2^ year^−1^; such rates rival rates from reefs with high coral cover in the Caribbean and the Indian Ocean (prior to bleaching in 2015‒2016; Ref.^15^). Reduced ocean temperatures from upwelling events in the GoP have buffered corals from recent thermal stress events, allowing the rapid recovery of these systems along with a further increase in coral cover that surpasses the baseline for Saboga reported for the last 50 years^67^. Consequently, reefs in the GoP now exhibit a high accretion potential^36,46^.

### Current and historical trends in reef-accretion potential

RAPs in the GoP are similar to those of reefs with moderate coral cover (~ 30–50%) in other regions, whereas accretion rates in the GoC are similar to those of degraded reefs elsewhere, which exhibit either little-to-no growth or net erosion. Our RAP estimates for the GoP (5.5 ± 0.3 mm year^−1^) are similar to accretion rates reported for reefs in Bonaire, Dutch Caribbean (average = 4.9 mm year^−1^; Ref.^12^) and inshore reefs of Pohnpei and Kosrae in the central Pacific (average = 5.9 mm year^−1^; Ref.^14^), but they fall below estimates for Palau and Yap in the western Pacific (average = 7.9 mm year^−1^; Ref.^15^). Although the reefs in the GoP exhibit coral-cover values comparable to high-coral-cover reefs (~ 50–70%) in other regions^12,15^, bioerosion pressure in the ETP is more than two-fold the bioerosion pressure that other regions exhibit (Supplementary Table S4).

**Figure 4:**
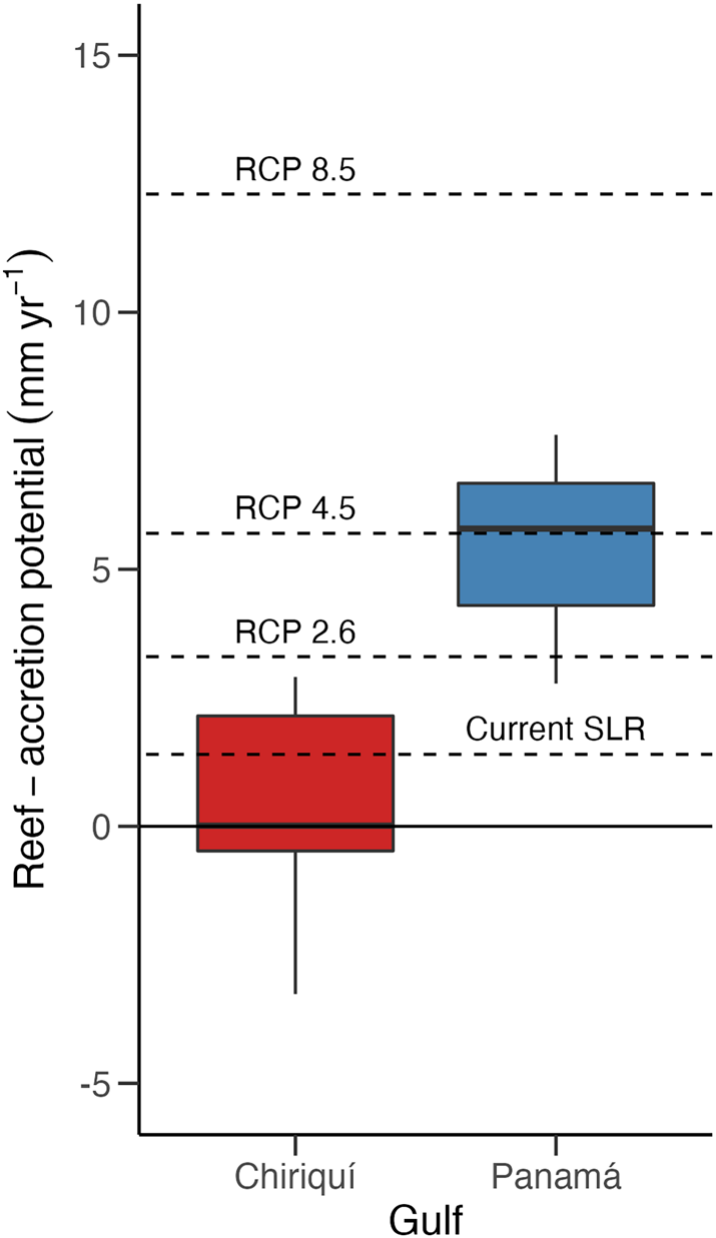
Boxplot of median (± interquartile range) reef-accretion potential (mm year^−1^) for each gulf during the most recent survey (spring 2018). The horizontal lines represent the projected mean sea-level rise for each of three RCPs^12^, as well as the current rate of sea-level rise for Panamá^74^. The black horizontal line delimits the division between net accretion and net erosion.

Our RAP estimates for the GoC (0.3 ± 0.5 mm year^−1^) are similar to contemporary accretion rates reported for degraded reefs in the ETP (0.07–0.29 mm year^−1^; Ref.^28^). These accretion rates are much lower than rates estimated for most other Indo-Pacific regions, which, like the Panamanian reefs, experienced significant coral mortality during the 2015–2016 ENSO event, including the Chagos Archipelago in the Indian Ocean (2.9 mm year^−1^, Ref.^12^) and the islands of Pohnpei, Kosrae and Majuro in the central Pacific (6 mm year^−1^; Ref.^14^). Accretion rates in the GoC currently resemble those of the Seychelles (post-bleaching, in 2017) and those of degraded Caribbean reefs (− 0.4 to 1.3 mm year^−1^; Ref.^12^). Like those locations, most reefs in the GoC cannot accrete rapidly enough to keep up with current rates of sea level rise in Panamá (1.4 mm year^−1^; Ref.^12^; Fig. 4).

The accretion rates required for reefs in both gulfs to keep up with sea level under all RCPs are higher than their millennial-scale accretion rates during periods of active reef growth over the last ~ 7000 years, which were 2.4 mm year^−1^ for Canales de Tierra in the GoC and 1.6 mm year^−1^ for Contadora in the GoP^42^. Compared with Holocene accretion rates, present-day estimates of RAP are lower in the GoC but higher in the GoP. The modern RAP rates, however, are estimates of the maximum vertical accretion that the reefs can achieve, assuming environmental conditions remain favorable for continuous reef growth. In contrast, observed reef-accretion rates are millennial-scale records that include stops and starts in reef growth caused by variations in environmental conditions through time, leading to accretion rates that are lower on millennial scales than on decadal to centennial scale^27^.

Our RAP estimates do not take into account future decreases in calcification rates and increases in bioerosion caused by future thermal-stress events and ocean acidification, which would decrease the observed accretion of these reefs, especially for the highly vulnerable GoC^71^. Furthermore, post-depositional compaction of the open *Pocillopora* framework over centennial to millennial timescales necessarily produces lower estimates of reef accretion during the Holocene compared with the accretion of contemporary in situ* Pocillopora* reef framework. *Pocillopora* corals have a branching skeleton that produces a highly porous framework when the corals are alive. The contemporary framework is readily broken down and compacted as new material is deposited above it^28^. Thus, meters of coral growth at ecological timescales translate to centimeters of reef accretion at geological timescales^72^.

Our calculations of accretion rates incorporated the density and porosity of the reef-framework (see Materials and Methods). When we exclude porosity from the RAP equation to estimate current, millennial-scale accretion rates, the GoC still exhibits lower accretion rates (0.7 mm year^−1^), whereas the GoP currently exhibits higher accretion rates (4.7 mm year^−1^) than those recorded for the Holocene (2.4 mm year^−1^ and 1.6 mm year^−1^, respectively). This result supports the hypothesis that recent warming has reversed the historical pattern of upwelling being less favorable for coral growth and reef accretion^42^: reef accretion is now more rapid in the strongly-upwelling GoP than it was over the last ~ 7000 years^36^.

### Coral-cover thresholds

The coral-cover thresholds for maintaining net-positive carbonate budgets for the GoP (43%) and the GoC (40%) are much higher than the threshold values predicted for the Caribbean (10%), the Chagos Archipelago (12%), and the western Pacific (~ 10–12%; Refs.^15,17,73^). Because of the high bioerosion pressure in the region, reefs in the ETP require very high coral cover (~ 75–80%) just to achieve net accretion rates similar to reefs with moderate levels of coral cover (~ 30–50%) in other regions (Supplementary Table S4). Eastern Pacific reefs that exhibit moderate levels of coral cover, such as the ones in the GoC, have low accretion-potential and are at risk of being tipped into a state of net erosion (Supplementary Table S4; Ref.^5^). The GoP requires 4% more coral cover than the GoC to keep up with predicted sea-level rise scenarios, due to the higher bioerosion rates^7^. Average coral cover in the GoC (39%) is currently below the 59% coral cover threshold required to keep up with sea level under the aggressive emissions-reduction scenario predicted under RCP 2.6. Total coral cover would have to increase to at least 75% for reefs there to keep pace with sea-level rise under RCP 4.5, and some reefs in the GoC are already experiencing net neutral accretion rates or net erosion (Fig. 4).

Given the predicted increase in thermal stress, which is expected to continue to reduce coral-calcification rates and coral cover, it is likely that reefs in the GoC will not be able to keep up with future sea-level rise even if emissions are reduced^36,54^. On the other hand, coral cover in the GoP (75%) is currently above the 61% coral-cover-threshold value to keep up with RCP 2.6 there and just below the 76% threshold value for RCP 4.5 (Fig. 4; Ref.^36^). Reefs in the GoP have the capacity to keep up with sea-level rise under moderate climate change scenarios if environmental conditions remain favorable. These reefs, however, remain highly vulnerable to disturbances that may slightly decrease coral cover because of the high levels of coral cover they require to maintain high RAP rates. The maximum RAPs that can be achieved by reefs in the GoC and the GoP, assuming 100% coral cover, are 9.7 mm year^−1^ and 9.4 mm year^−1^, respectively, indicating that reefs from both gulfs are incapable of producing enough calcium carbonate to keep up with the predicted rate of sea-level rise of 12.3 mm year^−1^ under RCP 8.5(Fig. 4; Ref.^36^).

It is important to note that our coral-cover thresholds are based on shallow reefs that are composed primarily of *Pocillopora*. In many cases, *Pocillopora* was the only coral taxon reported within our transects, and taxa with massive or sub-massive growth morphologies comprised a small fraction of total coral cover. Although the deeper assemblage of massive corals is more diverse than the *Pocillopora* assemblage we surveyed, massive corals usually exhibit lower levels of calcification than *Pocillopora* under the same environmental conditions^50^. The dominance of *Pocillopora* maximizes the calcification potential of Panamanian reefs, at least in the shallow reef-zones, and it demonstrates that functional diversity and assemblage variability do not play major roles in the calcification potential of shallow reef-zones of the ETP^50^.

### Future challenges to the resilience of eastern Pacific reefs

Environmental changes coupled with sea-level rise could have a strong influence on future reef-accretion potential. For instance, rising sea level could drive a significant decline in water quality by increasing terrigenous-sediment discharges into the gulfs, thereby increasing nutrient and turbidity levels^28,75,76^. Reduced light availability could cause a decrease in the growth-potential of coral assemblages and a shallowing of the depth-threshold for reef drowning^77^. This is particularly problematic for reefs in Pacific Panamá, which already experience high turbidity and light limitation due to large tidal ranges^23,26^. Similarly, high turbidity during the past 6000 years has been responsible for suppressing reef development in inshore regions of the southern Great Barrier Reef^78,79^. Moderate levels of turbidity, however, can also protect corals from marine heatwaves by buffering the stress that corals experience from high light intensity and promote recovery through heterotrophy^80,81^.

Long-term surveys have described eastern Pacific reefs as systems that are highly resilient to thermal stress^70,82^. Rapid recovery recorded on individual reefs following disturbances, combined with a lack of evidence for regional degradation have led to this consensus. Our results, however, provide evidence that environmental conditions are deteriorating at smaller, yet significant, spatial scales of hundreds of kilometers, and that continued decline of these conditions would threaten future reef development in the region. The GoC is currently warming at a faster rate than the GoP, where seasonal upwelling buffers thermal stress, and recurring thermal-stress events are predicted to become more intense and more frequent^19,36^. Although most coral species in the eastern Pacific have demonstrated resilience to recent thermal stress^70,83^, rising temperatures and recurring heat waves will likely continue to compromise coral calcification rates, reduce coral cover, and jeopardize the ability of reefs of the ETP to keep up with future sea-level rise^14,84^. This phenomenon has already been reported in the Red Sea, where increasing temperatures that do not exceed the bleaching threshold of corals are reducing rates of calcification and growth^84–86^. Corals may survive in small-scale refugia from thermal stress, allowing some individual reefs to recover rapidly^87,88^, and in the case of the GoP they may even support coral-reef development for some time.

Coral-reef ‘oases’ that possess relatively high coral cover and carbonate production, such as the GoP, provide a measure of optimism in the face of current, global trends of reef degradation^89,90^. This positivity should not distract attention from degrading reefs like those in the GoC^91,92^. Whereas reefs in the GoP may be able to keep pace with the moderate rates of sea-level rise projected under RCP 4.5, that projection involves cutting half of global greenhouse-gas emissions by 2080. Furthermore, the fact that none of Panamá’s reefs have the capacity to keep pace with sea-level rise projected under RCP 8.5 (12.3 mm year^−1^) suggests that aggressively mitigating greenhouse-gas emissions is essential to promoting the recovery of degraded reefs and persistence of even the most resilient coral reefs.

## Materials and methods

### Ecological surveys

As part of an associated study^36^, we performed ecological surveys at six sites, with three sites in each gulf (Fig. 1), during the spring and autumn seasons of 2016 and 2017, and during the spring season of 2018. We quantified the composition of the benthic assemblage within each site with six replicate, 25-m long transects placed haphazardly on the reef slope (~ 3 m below mean sea level [MSL]). The benthic composition of each transect was determined using the point-intercept method. SCUBA divers recorded the benthic component underlying the transect tape at each 25-cm mark, yielding 100 points per transect. Stony corals were identified to the genus or species level, and the rest of the benthic components were grouped into the broad categories of fine algal turfs, thick algal turfs, fleshy macroalgae, crustose coralline algae (CCA), rubble, and sand^36^. *Halimeda* spp. and other branching coralline algae were scarce at our sites and were not recorded in any of our transects.

### Calcification rates

*Pocillopora* colonies were collected and out-planted at experimental calcification monitoring stations at each site following the method detailed by Kuffner et al.^57^. Briefly, *Pocillopora* fragments were collected and deployed onto concrete blocks within each study site. Each fragment was epoxied to a polyvinyl chloride (PVC) base that was bolted onto a concrete block, with twenty fragments out-planted at each site^36^. Each fragment was photographed and its buoyant weight was measured at 6-month intervals beginning in spring 2016, autumn 2016, and spring 2017, and for 1 year from spring 2017 to spring 2018, yielding growth estimates for the wet and dry seasons as well as annual growth. Surface areas were estimated using top-down photos to calculate the planar surface area of the canopy for each fragment^93^.

We estimated the dry weight of each fragment using a non-invasive methodology that determines a coral’s dry skeletal weight based on its buoyant weight^56^. This methodology allowed corals to be weighed while they were submerged, preventing the death of the living tissue, and it allowed us to estimate changes in dry weight ($\Delta {W}_{a})$ across multiple growth periods. We estimated calcification rates of *Pocillopora* by standardizing the change in dry weight by the average surface area over the growth period^93^. For the remaining coral taxa that were recorded in our surveys, which on average accounted for ≤ 1% of coral cover, we used taxon-specific calcification rates reported in the literature for the eastern Pacific (Supplementary Table S5).

### Bioerosion rates

Bioerosion by cryptic macroborers and grazers was estimated using rates reported for the *Pocillopora* framework of Uva Island reef for the GoC, rates reported for Saboga Reef for the GoP^61,64^, and the estimates of benthic cover measured in this study:

1 $${MB}_{i}=\left({L}_{i}\times {ml}_{g}\right)+ \left({T}_{i}\times {mt}_{g}\right)+\left({D}_{i}\times {md}_{g}\right),$$

where ${MB}_{i}$ is the macroborer bioerosion rate of transect *i* (kg CaCO_3_ m^−2^ year^−1^), ${L}_{i}$ is the proportional coral cover, ${ml}_{g}$ is the bioerosion rate of borers on live coral (kg CaCO_3_ m^−2^ year^−1^) for gulf $g$, ${T}_{i}$ is the proportional cover of thick algal turfs in transect *i*, $m{t}_{g}$ is the bioerosion rate of borers on substrate covered with thick algal turfs (kg CaCO_3_ m^−2^ year^−1^) for gulf $g$, ${D}_{i}$ is the proportional dead substrate cover on transect *i*, and ${md}_{g}$ is the bioerosion rate of borers on dead substrate (kg CaCO_3_ m^−2^ year^−1^) for gulf $g$. Because there are currently no estimates for microbioerosion rates for the eastern Pacific^8^, we used the average microbioerosion rate from the Indo-Pacific ReefBudget database of 0.233 kg m^−2^ year^−1^ (Ref.^94^).

Physical bioerosion by excavating sponges is likely included in the cryptic macroboring rates; therefore, only chemical dissolution rates were estimated for sponge bioerosion. We used the chemical dissolution rates and the estimated prevalence of sponge infestation reported for the Mexican Pacific^95,96^. The prevalence of sponge infestation is the proportion of sampled coral colonies in which boring sponges were present:

2 $$S{B}_{i}= \left(\left({L}_{i}\times 0.46\right)\times 0.85\right)+\left(\left({RC}_{i}\times 0.46\right)\times 0.85\right)+\left(\left(F{C}_{i}\times 0.56\right)\times 0.85\right),$$

where $S{B}_{i}$ is the sponge-bioerosion rate (kg CaCO_3_ m^−2^ year^−1^) in transect *i*, ${L}_{i}$ is the proportional live coral cover determined for transect *i*, 0.46 is the average relative abundance of living corals in which boring sponges are present^96^, 0.85 kg CaCO_3_ m^−2^ year^−1^ is the average chemical dissolution rate for sponges in the eastern Pacific^95^, ${RC}_{i}$ is the proportional rubble cover reported for transect *i*, 0.46 is the average proportion of rubble fragments in which boring sponges are present^96^, ${FC}_{i}$ is the proportional cover of coral-reef framework (dead coral skeletons in growth position) reported for transect *i*, and 0.56 is the average proportion of framework substrate in which boring sponges are present^96^.

Estimates of bioerosion by the sea urchin *D. mexicanum* (Echinoidea), the only bioeroding sea urchin observed in our study, were calculated for each site. To estimate *D. mexicanum* density, six 25 × 1 m video belt-transects were haphazardly captured at each site for each sampling period by SCUBA divers with a GoPro camera, which was pointed down and positioned 1 m from the reef surface as they travelled along the transect at a constant speed (see “Sea-Urchin Densities and Bioerosion” in the Supplementary Methods). For sites in the GoC, bioerosion estimates for *D. mexicanum* were calculated using the individual sea-urchin bioerosion rates previously reported for Uva Island reef^64^. For sites in the GoP, bioerosion estimates for *D. mexicanum* were calculated using the individual urchin bioerosion rates previously reported for Saboga Reef^61^. For both gulfs, the bioerosion rates of *D. mexicanum* on live, dead, and algal-dominated *Pocillopora* framework were multiplied by the sea-urchin densities from the video transects:

3 $$U{B}_{i}=\left(\left({L}_{i}\times {U}_{i}\times {ul}_{g}\right)+\left({D}_{i}\times {U}_{i}\times {ud}_{g}\right)+ \left({T}_{i}\times {U}_{i}\times {ut}_{g}\right)\right)\times \left(\frac{365}{1000}\right),$$

where $U{B}_{i}$ is the total sea-urchin bioerosion rate for transect *i*, ${U}_{i}$ is the sea-urchin density for transect *i* (ind m^−2^), ${ul}_{g}$ is the mean bioerosion rate on live *Pocillopora* (g CaCO_3_ ind^−1^ day^−1^) for gulf $g$, ${ud}_{g}$ is the mean bioerosion rate on dead substrate (g CaCO_3_ ind^−1^ day^−1^) for gulf $g$, and ${ut}_{g}$ is the mean bioerosion rate on substrate covered by thick algal turfs (g CaCO_3_ ind^−1^ day^−1^) for gulf $g$. The data were transformed from g CaCO_3_ m^−2^ day^−1^ to kg CaCO_3_ m^−2^ year^−1^ by using the conversion factor (365/1000).

Bioerosion by parrotfish (Labridae) was estimated for each site using estimates of population density and known bioerosion rates^94,97^. To estimate fish population densities, six haphazardly placed 25 × 4 m belt transects were visually surveyed by SCUBA divers, who recorded every fish species encountered in the transect. Fish surveys were only performed during 2018 and 2019, so these estimates were extrapolated for the surveys from 2016 and 2017 (Ref.^98^; see “Parrotfish Grazing” in the Supplementary Methods). Two parrotfish species, *Scarus ghobban* and *S. rubroviolaceus*, were recorded at our sites. We estimated bioerosion rates using bite rates and estimates of substrate removal. We used bite rates reported for *S. ghobban* in the GoC and the GoP^97^. For *S. rubroviolaceus*, we used the average bite rates reported in the Indo-Pacific ReefBudget database. The variables determining substrate removal for both species—proportion of bites leaving scars and volume removed per bite—were obtained from the ReefBudget Indo-Pacific database, and the rates of bioerosion were then calculated using the ReefBudget parrotfish bioerosion equations^94^. Parrotfish-bioerosion rates were then multiplied by their respective species’ densities (ind m^−2^) to obtain parrotfish-bioerosion rates for each transect.

Rates of bioerosion by the corallivorous pufferfish *Arothron meleagris* (Tetraodontidae) were calculated using our fish-survey data and estimates of individual *A. meleagris* bioerosion rates previously reported for Gorgona Island, Colombia^99^:

4 $$A{B}_{i}=\left(\frac{{d}_{a}\times 365}{1000}\times {A}_{i}\right)\times 0.6,$$

where $A{B}_{i}$ is bioerosion by *A. meleagris* for transect *i*, ${d}_{a}$ is the destruction rate per fish (g CaCO_3_ ind^−1^ day^−1^), 365 and 1000 are conversion factors from g CaCO_3_ ind^−1^ day^−1^ to kg CaCO_3_ ind^−1^ year^−1^, ${A}_{i}$ is our recorded *A. meleagris* population density for transect *i* (ind m^−2^), and 0.6 is a correction factor for the proportion of fish actively feeding at a given time^97^.

We validated our survey-based estimates of sea-urchin and fish bioerosion by comparing the densities of those bioeroding taxa estimated in our study with those estimated in previous studies (see “Sea Urchin Densities and Bioerosion” and “Parrotfish Grazing” in the Supplemental Methods). Although the populations of *D. mexicanum*, *S. ghobban*, *S. rubroviolaceous*, and *A. meleagris* showed considerable variability in space and time, we found that our density estimates were generally similar to those determined in previous studies (Supplementary Tables S7 and S8; Supplementary Figs. S2–S5), suggesting that our estimates of external macrobioerosion are robust.

### Carbonate-budget model

Gross carbonate production was estimated using a modified version of the Indo-Pacific ReefBudget methodology^94^. We multiplied the relative abundances of calcifying taxa recorded along each point-intercept transect by their taxon-specific calcification rates. Since *Pocillopora* are the prevalent reef-building corals of the ETP and they were the dominant corals at our sites, we estimated in situ calcification rates for individual *Pocillopora* colonies. Although we calculated seasonal calcification rates for *Pocillopora* in each gulf, we used annual calcification rates for each gulf averaged across the entire dataset (GoC = 2.08 g CaCO_3_ cm^−2^ year^−1^; GoP = 2.23 g CaCO_3_ cm^−2^ year^−1^) to calculate carbonate-production rates because they are, by convention, annual estimates (kg CaCO_3_ m^−2^ year^−1^).

The sum of the gross carbonate production for each coral taxon and crustose coralline algae yielded the gross carbonate production for each transect in kg CaCO_3_ m^−2^ year^−1^. Total bioerosion for transect *i*, ${TB}_{i}$, was calculated as the sum of the bioerosion rates attributable to macroboring, microboring, sponge dissolution, *D. mexicanum*, parrotfish, and *A. meleagris*. We then estimated net carbonate production (kg CaCO_3_ m^−2^ year^−1^) by subtracting the bioerosion rates from the gross calcium-carbonate production rates in each transect.

### Reef-accretion potential (RAP)

To convert rates of net calcium-carbonate production (kg CaCO_3_ m^−2^ year^−2^) into estimates of vertical accretion (mm year^−1^), we accounted for reef-framework porosity using the compaction rates estimated for reef-framework cores taken at each site^100^, where porosity is equal to one minus the compaction rate (Supplementary Table S6). The average, overall framework porosity was estimated for each core excluding the first two meters of surface framework because this top-most interval represented the contemporary, open framework accumulation that was not yet packed in sediment, and we were interested in estimating long-term accretion rates which must account for compaction:

5 $${\rho }_{x}=\left({C}_{x}\times {D}_{f}\right)+\left({\phi }_{x}\times {D}_{w}\right),$$

where ${\rho }_{x}$ is the corrected framework density for site *x* (g cm^−3^), ${C}_{x}$ is the average compaction rate of the cores extracted at site *x* (see Supplementary Table S6 for a description of the calculation of ${C}_{x}$), ${D}_{f}$ is the framework density calculated from *Pocillopora* skeletons (g cm^−3^), ${\phi }_{x}$ is the average porosity calculated from the cores extracted at site *x*, and ${D}_{w}$ is the average density of seawater (g cm^−3^).

To estimate the framework density (${D}_{f}$), we assumed that the reef framework was composed primarily of dead *Pocillopora* skeletons, based on palaeoecological records^27,42^. We used the dead skeletons of the *Pocillopora* fragments from the calcification experiments (see Calcification Rates above) to calculate the average skeletal density within each gulf. We oven-dried the skeletons for 24 h at 60 °C, measured their dry weights, and dipped them quickly into paraffin wax at 110–115 °C. The waxed skeletons were submerged into a graduated cylinder with deionized water to estimate the bulk volume of the skeleton based on the volume of displaced water. There was no significant difference in skeletal density between gulfs (F_1,23_ = 0.009, p = 0.934), so the overall average density (1.84 g cm^−3^; SD =  ± 0.20) was used for the substrate density value (${D}_{f}$).

We then divided the net carbonate production rate (kg CaCO_3_ m^−2^ year^−1^) by the framework density and added the estimated contribution of sediments to estimate the reef-accretion potential^17^:

6 $$RA{P}_{i}=\left(\frac{{G}_{i}}{{\rho }_{x}}+S\right)\times 1000,$$

where $RA{P}_{i}$ is reef-accretion potential (mm year^−1^) for transect *i*, ${G}_{i}$ is net carbonate production for transect *i*, $S$ is the proportion of allochthonous and autochthonous sediments that are incorporated into the framework and contribute to reef accretion (see “Sedimentation Rates” in the Supplementary Methods), ${\rho }_{x}$ is the framework density at site *x* corrected for porosity (g cm^−3^), and 1000 is a conversion factor from kg CaCO_3_ m^−2^ year^−1^ to mm year^−1^.

We note that reef-accretion potential is likely a conservatively high estimate of true reef-accretion rate as budget-based estimates of net carbonate production only quantify the biological processes that contribute to reef accretion and do not account for physical or chemical erosion (cf.^94^). On the other hand, reef rugosity was not quantified at our sites because our benthic surveys were not initially intended for estimating carbonate budgets. Estimating carbonate-production rates based on the flat surface of a transect underestimates the surface area available for calcification, whereas incorporating rugosity into a carbonate-budget model accounts for the three-dimensional nature of reefs (but see Supplementary Methods). By not including rugosity in our estimates, we may, therefore, be underestimating the rates of gross carbonate production and bioerosion at our sites. Although these sources of uncertainty affect the precision of carbonate production and RAP rates, the magnitude of the between-gulf differences suggests that the overarching trends are robust.

### Data analysis

We used linear mixed-effects models to evaluate spatial and temporal differences in annual and seasonal coral calcification, gross carbonate production, bioerosion, and net carbonate production. Differences in annual *Pocillopora* calcification rates between gulfs were assessed with sites modeled as random effects. To assess the differences in seasonal *Pocillopora* calcification rates, we treated site and season as fixed factors and used the identity of the experimental coral out-plant (‘coral ID’) as a random factor. Designating coral ID as a random factor allowed us to include corals of varying ages (i.e., from different out-planting dates) in a repeated-measures design. Differences in gross carbonate production, bioerosion, and net carbonate production were evaluated with gulfs and time intervals as fixed effects, and sites modeled as random effects. Residual plots were visually inspected for overfitting and for deviations from normality and homoscedasticity. We also assessed the possibility of spatial autocorrelation within different time intervals for each model by plotting autocorrelation-corrected residuals using the autocorrelation function from the “nlme” R package^101^. Using the raw data resulted in the best model-fits for calcification data, net calcium-carbonate production, and gross calcium-carbonate production. For bioerosion, the log_10_-transformed data provided the best model-fit.

We also used linear mixed-effect models to determine the threshold value of coral cover that each gulf required to maintain a positive carbonate budget (kg CaCO_3_ m^−2^ year^−1^) and to keep up with future sea-level rise projected by the IPCC^10^ for RCPs 2.6 (4 mm year^−1^), 4.5 (7 mm year^−1^), and 8.5 (15 mm year^−1^). RCP 2.6 is an aggressive-mitigation scenario, which predicts that greenhouse-gas emissions will begin to decline by 2020 and reach 0 by 2100, limiting warming to 1.5 °C. RCP 4.5 is moderate-mitigation scenario, which predicts that greenhouse-gas emissions will peak in 2040 and decline by ~ 50% by 2080, likely limiting warming to 2 °C. RCP 8.5 is a business-as-usual scenario that assumes no reductions in greenhouse-gas emissions and continuous warming^74^. To estimate the localized rate of sea-level rise for Pacific Panamá, tide-gauge data from the Permanent Service for Mean Sea Level were retrieved for Balboa, Panamá^102,103^. We used current, localized trends in sea-level rise to predict future rates of sea-level rise for Panamá. By comparing the differences in magnitude between the global average sea-level rise and Panamá’s localized sea-level rise, we applied correction factors to the global rates of sea-level rise projected for RCPs 2.6, 4.5 and 8.5 to estimate the localized rates for these scenarios.

We used the data from spring 2018 to approximate the current, ecological state of Panamá’s reefs. Net carbonate production and RAP were used as the response variables for their respective models, with coral cover and gulf as predictor-variables for both models. The raw data for both net carbonate production and RAP provided the models with the best fit. Residual plots were visually inspected for overfitting and for deviations from normality and homoscedasticity. All models were developed in R version 4.2.2 (Ref.^39^) using the lme function from the “nlme” package^101^.

## Supplementary Information

Supplementary Information.

## Data Availability

The datasets for fish and sea-urchin populations, coral calcification and skeletal density, benthic surveys, and carbonate production are archived and publicly available in the Biological and Chemical Oceanography Data Management Office (https://www.bco-dmo.org/project/655899).
