# Supplementary material for: Upwelling, climate change, and the shifting geography of coral reef development
Source: Sci Rep. 2023 Feb 7;13:1770. doi: 10.1038/s41598-023-28489-0 (PMC9905564; doi:10.1038/s41598-023-28489-0)
Supplement: Supplementary file 1 — Supplementary Information. [file 41598_2023_28489_MOESM1_ESM.docx]

*Scientific Reports*

Supplementary Information for

**Upwelling, climate change, and the shifting geography of coral reef development**

Victor Rodriguez-Ruano^1^, Lauren T. Toth^2^, Ian C. Enochs^3^, Carly J. Randall^4^, Richard B. Aronson^1^

*^1^ Department of Ocean Engineering and Marine Sciences, Florida Institute of Technology, 150 West University Boulevard, Melbourne, Florida 32901, USA*

*^2^ St. Petersburg Coastal and Marine Science Center, U.S. Geological Survey, 600 4^th^ St. South, St. Petersburg, Florida 33701, USA*

*^3^ NOAA, Atlantic Oceanographic and Meteorological Laboratory, Ocean Chemistry and Ecosystem Division, 4301 Rickenbacker Cswy., Miami, Florida 33149, USA*

*^4^ Australian Institute of Marine Science, PMB No. 3, Townsville, Queensland 4810, Australia*

**Supplementary Information included in this file:**

Supplementary Tables S1–S8

Supplementary Methods

Supplementary Figures S1–S5

***Any use of trade, firm, or product names is for descriptive purposes only and does not imply endorsement by the U.S. Government.***

**SUPPLEMENTARY INFORMATION**

**Table S1:** Best-fit linear mixed-effect models developed to predict the threshold values of percent coral cover required for reefs in each gulf to maintain a positive rate of net carbonate production, and to maintain accretion rates high enough to keep up with different scenarios of sea-level rise. SE, standard error; df, degrees of freedom.

| **Net carbonate production** | | | | | |
| --- | --- | --- | --- | --- | --- |
|  | **Value** | **SE** | **df** | **t-value** | **p** |
| **Intercept** | -9.35 | 0.40 | 28 | -23.65 | <0.001 |
| **Gulf** | -0.71 | 0.39 | 4 | -1.82 | 0.08 |
| **Coral cover** | 0.23 | 0.009 | 28 | 25.17 | <0.001 |
| **Reef accretion potential** | | | | | |
|  | **Value** | **SE** | **df** | **t-value** | **p** |
| **Intercept** | -5.770 | 0.273 | 28 | -21.104 | <0.0001 |
| **Gulf** | -0.307 | 0.271 | 4 | -1.135 | 0.265 |
| **Coral cover** | 0.155 | 0.007 | 28 | 23.626 | <0.0001 |

**Table S2:** Bioerosion estimates from Eakin’s^7^ carbonate budget model for Uva Reef, Panama, and the relative contribution of infaunal erosion to total reef bioerosion. All bioerosion estimates are in kg CaCO_3_ m^-2^ yr^-1^.

| **Reef Zone** | ***Diadema*** | **Fish** | **Infauna** | **Corallivores** | **Total** | **Contribution of infaunal erosion (%)** | **Reference** |
| --- | --- | --- | --- | --- | --- | --- | --- |
| Back reef | 0.08 | 0.02 | 6.29 | 0.02 | 6.41 | 98% | ^1^ |
| Reef flat | 0.01 | 1.15 | 3.67 | 0.00 | 4.83 | 76% | ^1^ |
| Fore reef | 1.04 | 1.28 | 5.95 | 0.02 | 8.29 | 72% | ^1^ |
| Reef base | 4.38 | 1.25 | 8.01 | 0.00 | 13.64 | 59% | ^1^ |
| Fore-reef slope (Spring 2016) | 0.02 | 0.18 | 5.69 | 0.09 | 5.98 | 95% | This study |
| Fore-reef slope (Spring 2018) | 0.33 | 0.18 | 6.65 | 0.09 | 7.25 | 92% | This study |

**Table S3:** Carbonate budget outputs for each site in each time interval.

| **Time** | **Site** | **Mean (± SE) gross carbonate production (kg m^-2^ yr^-1^)** | **Mean (± SE) bioerosion (kg m^-2^ yr^-1^)** | **Mean (± SE) net carbonate production (kg m^-2^ yr^-1^)** | **Mean (± SE) RAP (mm yr^-1^)** |
| --- | --- | --- | --- | --- | --- |
| Spring 2016 | Canales | 6.7 ± 1.2 | -6.4 ± 0.4 | 0.4 ± 1.5 | 0.2 ± 1.0 |
| Spring 2016 | Coiba | 13.0 ± 2.6 | -6.5 ± 0.4 | 6.5 ± 2.9 | 4.2 ± 1.8 |
| Spring 2016 | Uva | 11.6 ± 1.4 | -6.6 ± 0.2 | 5.0 ± 1.6 | 3.3 ± 1.0 |
| Spring 2016 | Contadora | 19.3 ± 0.7 | -5.5 ± 0.2 | 13.9 ± 0.7 | 9.4 ± 0.5 |
| Spring 2016 | Pedro Gonzalez | 17.5 ± 0.9 | -5.2 ± 0.1 | 12.3 ± 1.0 | 8.3 ± 0.6 |
| Spring 2016 | Saboga | 17.6 ± 1.0 | -5.7 ± 0.1 | 11.9 ± 1.0 | 7.5 ± 0.6 |
| Autumn 2016 | Canales | 8.7 ± 0.8 | -6.3 ± 0.2 | 2.3 ± 1.0 | 1.5 ± 0.6 |
| Autumn 2016 | Coiba | 13.0 ± 1.8 | -5.4 ± 0.4 | 7.6 ± 1.6 | 4.9 ± 1.0 |
| Autumn 2016 | Uva | 12.2 ± 1.1 | -6.0 ± 0.4 | 6.1 ± 1.5 | 4.0 ± 0.9 |
| Autumn 2016 | Contadora | 15.9 ± 1.4 | -6.4 ± 0.4 | 9.4 ± 1.6 | 6.6 ± 1.0 |
| Autumn 2016 | Pedro Gonzalez | 18.8 ± 0.5 | -5.2 ± 0.1 | 13.6 ± 0.6 | 9.3 ± 0.4 |
| Autumn 2016 | Saboga | 17.4 ± 0.7 | -6.4 ± 0.4 | 11.0 ± 0.8 | 7.1 ± 0.4 |
| Spring 2017 | Canales | 8.8 ± 1.3 | -7.5 ± 0.3 | 1.4 ± 1.5 | 0.9 ± 1.0 |
| Spring 2017 | Coiba | 11.1 ± 1.0 | -7.5 ± 0.3 | 3.6 ± 1.2 | 2.4 ± 0.7 |
| Spring 2017 | Uva | 11.1 ± 0.8 | -7.1 ± 0.2 | 4.0 ± 0.9 | 2.7 ± 0.6 |
| Spring 2017 | Contadora | 18.3 ± 1.1 | -5.7 ± 0.2 | 12.7 ± 1.1 | 8.6 ± 0.8 |
| Spring 2017 | Pedro Gonzalez | 14.8 ± 0.5 | -6.4 ± 0.1 | 8.3 ± 0.5 | 5.7 ± 0.4 |
| Spring 2017 | Saboga | 17.1 ± 0.5 | -5.9 ± 0.2 | 11.2 ± 0.5 | 7.1 ± 0.3 |
| Autumn 2017 | Canales | 9.8 ± 1.5 | -7.3 ± 0.3 | 2.6 ± 1.8 | 1.7 ± 1.1 |
| Autumn 2017 | Coiba | 11.4 ± 1.3 | -7.5 ± 0.3 | 3.9 ± 1.4 | 2.6 ± 0.9 |
| Autumn 2017 | Uva | 13.1 ± 0.6 | -7.2 ± 0.2 | 5.9 ± 0.7 | 4.0 ± 0.5 |
| Autumn 2017 | Contadora | 19.7 ± 0.7 | -6.0 ± 0.4 | 13.7 ± 0.8 | 9.4 ± 0.5 |
| Autumn 2017 | Pedro Gonzalez | 17.1 ± 0.8 | -6.1 ± 0.2 | 11.0 ± 1.0 | 7.5 ± 0.6 |
| Autumn 2017 | Saboga | 16.0 ± 0.7 | -7.1 ± 0.2 | 8.9 ± 0.9 | 5.9 ± 0.5 |
| Spring 2018 | Canales | 6.6 ± 1.2 | -8.0 ± 0.3 | -1.4 ± 1.5 | -0.9 ± 1.0 |
| Spring 2018 | Coiba | 9.0 ± 0.7 | -8.5 ± 0.5 | 0.5 ± 0.9 | 0.6 ± 0.5 |
| Spring 2018 | Uva | 8.9 ± 1.0 | -8.0 ± 0.3 | 0.9 ± 1.2 | 0.8 ± 0.8 |
| Spring 2018 | Contadora | 18.4 ± 0.3 | -8.9 ± 0.2 | 9.5 ± 0.3 | 6.5 ± 0.2 |
| Spring 2018 | Pedro Gonzalez | 16.8 ± 0.6 | -8.6 ± 0.1 | 8.1 ± 0.6 | 5.5 ± 0.4 |
| Spring 2018 | Saboga | 14.8 ± 0.6 | -9.2 ± 0.1 | 5.6 ± 0.6 | 3.7 ± 0.4 |

**Table S4:** Average gross carbonate production, bioerosion and net carbonate production for the two gulfs surveyed in this study, along with the estimates reported by other studies for reefs from different regions.

| **Study** | **Site** | **Gross CaCO_3_ production (kg CaCO_3_ m^-2^ yr^-1^)** | **Bioerosion (kg CaCO_3_ m^-2^ yr^-1^)** | **Net CaCO_3_ production (kg CaCO_3_ m^-2^ yr^-1^)** |
| --- | --- | --- | --- | --- |
| This study | Gulf of Panamá, spring 2018 | 16.7 | -8.9 | 7.8 |
| This study | Gulf of Chiriquí, spring 2018 | 8.1 | -8.1 | 0.0 |
| ^1^ | Uva Reef, 1992 | 4.6 | -8.3 | -3.7 |
| ^2^ | Maldives Archipelago, Indian Ocean | 8.3 | -3.4 | 4.9 |
| ^3^ | Palau, western Pacific Ocean | 10.3 | -0.3 | 10.0 |
| ^2^ | Mexican Caribbean | 3.2 | -3.4 | -0.2 |

**Table S5:** Mean calcification rates used to estimate carbonate production for the non-pocilloporid coral taxa encountered at our study sites. We used local calcification rates for the Gulf of Panamá and the Gulf of Chiriquí when available. We used average calcification rates from other localities of the ETP for taxa for which local calcification rates were not available.

| **Species** | **Locality** | **Site** | **Mean Density (g cm^-2^)** | **Mean extension rate (cm yr^-1^)** | **Mean Calcification rate (g cm^-2^ yr^-1^)** | **Reference** |
| --- | --- | --- | --- | --- | --- | --- |
| *Gardineroseris planulata* | Panamá | Uva Reef, Gulf of Chiriquí | 1.63 | 0.61 | 0.98 | ^4^ |
| *Pavona gigantea* | Panamá | Uva Reef, Gulf of Chiriquí | 1.48 | 0.92 | 1.35 | ^4^ |
| *Pavona gigantea* | Panamá | Saboga Reef, Gulf of Panamá | 1.75 | 0.85 | 1.49 | ^5^ |
| *Pavona varians* | Panamá | Uva Reef, Gulf of Chiriquí | 1.96 | 0.32 | 0.63 | ^4^ |
| *Porites lobata* | México | Marieta Islands | 1.19 | 0.56 | 0.67 | ^6^ |
| *Porites lobata* | México | Zacatoso, Oaxaca | 1.2 | 0.6 | 0.72 | ^7^ |
| *Porites panamensis* | México | Bahía de Los Angeles | 0.91 | 0.5 | 0.46 | ^8^ |
| *Porties panamensis* | México | Bahía de La Paz | 0.95 | 1.2 | 1.14 | ^8^ |
| *Porites panamensis* | México | Cabo Pulmo | 1.35 | 0.91 | 1.23 | ^9^ |
| *Porites panamensis* | México | Marieta Islands | 1.28 | 0.38 | 0.49 | ^9^ |
| *Porites panamensis* | México | La Entrega, Oaxaca | 1.12 | 0.31 | 0.35 | ^7^ |

**Table S6:** *In situ* measurements of the reef framework taken during push-coring operations at each site. Aluminum tubes, which were 7.6 cm in diameter, were forced by hand vertically into the uncemented frameworks^10^. Penetration was calculated by measuring the outer length of the core tube standing partially inserted into the reef, and subtracting that measurement from its total length. Recovery was measured by dropping a weighted measuring tape into the core barrel until it landed on the material collected and stopped descending. The inner length was subtracted from the total length of the tube to calculate recovery. Recovery as a proportion of penetration yielded compaction, and porosity was estimated as 1 minus compaction.

| **Gulf** | **Site** | **Core** | **Total Length**  **(cm)** | **Outer Length**  **(cm)** | **Inner Length**  **(cm)** | **Interval Penetration** | **Interval Recovery** | **Interval Compaction** | **Porosity** |
| --- | --- | --- | --- | --- | --- | --- | --- | --- | --- |
| Panamá | Contadora | EP08-24 | 474 | 328 | 397 | 146 | 77 | 0.53 | 0.47 |
| Panamá | Contadora | EP08-24 | 474 | 254 | 343 | 74 | 54 | 0.73 | 0.27 |
| Panamá | Contadora | EP08-24 | 474 | 169 | 298 | 85 | 45 | 0.53 | 0.47 |
| Panamá | Contadora | EP08-24 | 474 | 91 | 243 | 78 | 55 | 0.71 | 0.29 |
| Panamá | Contadora | EP08-25 | 494 | 397 | 459 | 97 | 35 | 0.36 | 0.64 |
| Panamá | Contadora | EP08-25 | 494 | 315 | 413 | 82 | 46 | 0.56 | 0.44 |
| Panamá | Contadora | EP08-25 | 494 | 250 | 365 | 65 | 48 | 0.74 | 0.26 |
| Panamá | Contadora | EP08-25 | 494 | 154 | 301 | 96 | 64 | 0.67 | 0.33 |
| Panamá | Contadora | EP08-25 | 494 | 79 | 251 | 75 | 50 | 0.67 | 0.33 |
| Panamá | Contadora | EP08-26 | 488 | 386 | 448 | 102 | 40 | 0.39 | 0.61 |
| Panamá | Contadora | EP08-26 | 488 | 333 | 422 | 53 | 26 | 0.49 | 0.51 |
| Panamá | Contadora | EP08-26 | 488 | 295 | 410 | 38 | 12 | 0.32 | 0.68 |
| Panamá | Contadora | EP08-26 | 488 | 209 | 375 | 86 | 35 | 0.41 | 0.59 |
| Panamá | Contadora | EP08-26 | 488 | 199 | 335 | 90 | 40 | 0.44 | 0.56 |
| Panamá | Contadora | EP09-27 | 476 | 380 | 423 | 96 | 53 | 0.55 | 0.45 |
| Panamá | Contadora | EP09-27 | 476 | 297 | 367 | 83 | 56 | 0.67 | 0.33 |
| Panamá | Contadora | EP09-27 | 476 | 249 | 336 | 48 | 31 | 0.65 | 0.35 |
| Panamá | Contadora | EP09-27 | 476 | 192 | 313 | 57 | 23 | 0.40 | 0.60 |
| Panamá | Contadora | EP09-27 | 476 | 91 | 253 | 101 | 60 | 0.59 | 0.41 |
| Panamá | Contadora | EP08-28 | 613 | 428 | 505 | 185 | 108 | 0.58 | 0.42 |
| Panamá | Contadora | EP08-28 | 613 | 372 | 471 | 56 | 34 | 0.61 | 0.39 |
| Panamá | Contadora | EP08-28 | 613 | 269 | 402 | 103 | 69 | 0.67 | 0.33 |
| Panamá | Contadora | EP08-28 | 613 | 201 | 345 | 68 | 57 | 0.84 | 0.16 |
| Panamá | Contadora | EP08-28 | 613 | 171 | 329 | 30 | 16 | 0.53 | 0.47 |
| Chiriquí | Canales | EP10-35 | 613 | 527 | 580 | 86 | 33 | 0.38 | 0.62 |
| Chiriquí | Canales | EP10-35 | 613 | 339 | 470 | 188 | 110 | 0.59 | 0.41 |
| Chiriquí | Canales | EP10-35 | 613 | 282 | 433 | 57 | 37 | 0.65 | 0.35 |
| Chiriquí | Canales | EP10-35 | 613 | 183 | 373 | 99 | 60 | 0.61 | 0.39 |
| Chiriquí | Canales | EP10-35 | 613 | 108 | 303 | 75 | 70 | 0.93 | 0.07 |
| Chiriquí | Canales | EP07-41 | 613 | 529 | 534 | 84 | 79 | 0.94 | 0.06 |
| Chiriquí | Canales | EP07-41 | 613 | 402 | 503 | 127 | 31 | 0.24 | 0.76 |
| Chiriquí | Canales | EP07-41 | 613 | 267 | 407 | 135 | 96 | 0.71 | 0.29 |
| Chiriquí | Canales | EP07-41 | 613 | 190 | 354 | 77 | 53 | 0.69 | 0.31 |
| Chiriquí | Canales | EP07-41 | 613 | 109 | 294 | 81 | 60 | 0.74 | 0.26 |
| Chiriquí | Canales | EP07-42 | 613 | 308 | 390 | 305 | 223 | 0.73 | 0.27 |
| Chiriquí | Canales | EP07-42 | 613 | 203 | 312 | 105 | 78 | 0.74 | 0.26 |
| Chiriquí | Canales | EP07-42 | 613 | 93 | 233 | 110 | 79 | 0.72 | 0.28 |
| Chiriquí | Canales | EP07-420 | 609 | 377 | 457 | 232 | 152 | 0.66 | 0.34 |
| Chiriquí | Canales | EP07-420 | 609 | 303 | 403 | 74 | 54 | 0.73 | 0.27 |
| Chiriquí | Canales | EP07-420 | 609 | 105 | 268 | 198 | 135 | 0.68 | 0.32 |
| Panamá | Pedro Gonzalez | EP17-101 | 605 | 383 | 580 | 222 | 25 | 0.11 | 0.89 |
| Panamá | Pedro Gonzalez | EP17-101 | 605 | 325 | 545 | 58 | 35 | 0.60 | 0.40 |
| Panamá | Pedro Gonzalez | EP17-101 | 605 | 286 | 531 | 39 | 14 | 0.36 | 0.64 |
| Panamá | Pedro Gonzalez | EP17-102 | 486 | 323 | 457 | 163 | 29 | 0.18 | 0.82 |
| Panamá | Pedro Gonzalez | EP17-102 | 486 | 259 | 411 | 64 | 46 | 0.72 | 0.28 |
| Panamá | Pedro Gonzalez | EP17-102 | 486 | 197 | 359 | 62 | 52 | 0.84 | 0.16 |
| Panamá | Pedro Gonzalez | EP17-102 | 486 | 149 | 343 | 48 | 16 | 0.33 | 0.67 |
| Panamá | Pedro Gonzalez | EP17-102 | 486 | 138 | 333 | 11 | 10 | 0.91 | 0.09 |
| Panamá | Pedro Gonzalez | EP17-103 | 604 | 305 | 487 | 299 | 117 | 0.39 | 0.61 |
| Panamá | Pedro Gonzalez | EP17-103 | 604 | 254 | 445 | 51 | 42 | 0.82 | 0.18 |
| Panamá | Pedro Gonzalez | EP17-104 | 477 | 350 | 407 | 127 | 70 | 0.55 | 0.45 |
| Panamá | Pedro Gonzalez | EP17-104 | 477 | 215 | 322 | 135 | 85 | 0.63 | 0.37 |
| Panamá | Pedro Gonzalez | EP17-104 | 477 | 201 | 319 | 14 | 3 | 0.21 | 0.79 |
| Panamá | Pedro Gonzalez | EP17-104 | 477 | 188 | 317 | 13 | 2 | 0.15 | 0.85 |
| Panamá | Saboga | EP09-30 | 497 | 325 | 420 | 172 | 77 | 0.45 | 0.55 |
| Panamá | Saboga | EP09-30 | 497 | 249 | 363 | 76 | 57 | 0.75 | 0.25 |
| Panamá | Saboga | EP09-31 | 489 | 394 | 450 | 95 | 39 | 0.41 | 0.59 |
| Panamá | Saboga | EP09-31 | 489 | 319 | 396 | 75 | 54 | 0.72 | 0.28 |
| Panamá | Saboga | EP09-31 | 489 | 229 | 329 | 90 | 67 | 0.74 | 0.26 |
| Panamá | Saboga | EP09-31 | 489 | 155 | 275 | 74 | 54 | 0.73 | 0.27 |
| Panamá | Saboga | EP09-31 | 489 | 122 | 249 | 33 | 26 | 0.79 | 0.21 |
| Panamá | Saboga | EP09-31 | 489 | 104 | 235 | 18 | 14 | 0.78 | 0.22 |
| Panamá | Saboga | EP09-32 | 493 | 403 | 464 | 90 | 29 | 0.32 | 0.68 |
| Panamá | Saboga | EP09-32 | 493 | 310 | 408 | 93 | 56 | 0.60 | 0.40 |
| Panamá | Saboga | EP09-32 | 493 | 224 | 344 | 86 | 64 | 0.74 | 0.26 |
| Panamá | Saboga | EP09-32 | 493 | 130 | 273 | 94 | 71 | 0.76 | 0.24 |
| Panamá | Saboga | EP09-33 | 468 | 382 | 439 | 86 | 29 | 0.34 | 0.66 |
| Panamá | Saboga | EP09-33 | 468 | 289 | 383 | 93 | 56 | 0.60 | 0.40 |
| Panamá | Saboga | EP09-33 | 468 | 215 | 327 | 74 | 56 | 0.76 | 0.24 |
| Panamá | Saboga | EP09-33 | 468 | 106 | 249 | 109 | 78 | 0.72 | 0.28 |
| Chiriquí | Coiba | EP13-53 | 604 | 434 | 460 | 170 | 144 | 0.85 | 0.15 |
| Chiriquí | Coiba | EP14-56 | 477 | 244 | 448 | 233 | 29 | 0.12 | 0.88 |
| Chiriquí | Coiba | EP14-56 | 477 | 212 | 423 | 32 | 25 | 0.78 | 0.22 |
| Chiriquí | Coiba | EP14-56 | 477 | 132 | 375 | 80 | 48 | 0.60 | 0.40 |
| Chiriquí | Coiba | EP14-56 | 477 | 57 | 310 | 75 | 65 | 0.87 | 0.13 |
| Chiriquí | Coiba | EP14-57 | 604 | 435 | 520 | 169 | 84 | 0.50 | 0.50 |
| Chiriquí | Uva | EP10-36 | 613 | 254 | 570 | 359 | 43 | 0.12 | 0.88 |
| Chiriquí | Uva | EP10-37 | 613 | 385 | 570 | 228 | 43 | 0.19 | 0.81 |
| Chiriquí | Uva | EP10-37 | 613 | 338 | 565 | 47 | 5 | 0.11 | 0.89 |
| Chiriquí | Uva | EP10-37 | 613 | 260 | 550 | 78 | 15 | 0.19 | 0.81 |
| Chiriquí | Uva | EP10-37 | 613 | 175 | 462 | 85 | 88 | 1.04 | -0.04 |
| Chiriquí | Uva | EP10-37 | 613 | 85 | 390 | 90 | 72 | 0.80 | 0.20 |
| Chiriquí | Uva | EP10-38 | 613 | 457 | 520 | 156 | 93 | 0.60 | 0.40 |
| Chiriquí | Uva | EP10-38 | 613 | 329 | 425 | 128 | 95 | 0.74 | 0.26 |
| Chiriquí | Uva | EP10-38 | 613 | 237 | 354 | 92 | 71 | 0.77 | 0.23 |
| Chiriquí | Uva | EP10-38 | 613 | 163 | 278 | 74 | 76 | 1.03 | -0.03 |
| Chiriquí | Uva | EP10-38 | 613 | 127 | 270 | 36 | 8 | 0.22 | 0.78 |
| Chiriquí | Uva | EP10-38 | 613 | 120 | 260 | 7 | 10 | 1.43 | -0.43 |
| Chiriquí | Uva | EP11-44 | 613 | 500 | 580 | 113 | 33 | 0.29 | 0.71 |
| Chiriquí | Uva | EP11-44 | 613 | 350 | 489 | 150 | 91 | 0.61 | 0.39 |
| Chiriquí | Uva | EP11-44 | 613 | 290 | 453 | 60 | 36 | 0.60 | 0.40 |
| Chiriquí | Uva | EP11-44 | 613 | 195 | 400 | 95 | 53 | 0.56 | 0.44 |
| Chiriquí | Uva | EP11-44 | 613 | 135 | 372 | 60 | 28 | 0.47 | 0.53 |
| Chiriquí | Uva | EP11-45 | 613 | 475 | 590 | 138 | 23 | 0.17 | 0.83 |
| Chiriquí | Uva | EP11-45 | 613 | 294 | 483 | 181 | 107 | 0.59 | 0.41 |
| Chiriquí | Uva | EP11-45 | 613 | 122 | 370 | 172 | 113 | 0.66 | 0.34 |

**Supplementary Methods**

**Benthic Rugosity**

Previous studies have measured the rugosity of the reef framework by draping a chain of known length over the framework and calculating the ratio between the known chain length and the linear distance covered by the draped chain^11^. Other studies that have not directly measured rugosity have used the published average rugosities of individual coral colonies to correct the calculated rates of carbonate production^12,13^. A comparison of framework-scale rugosity, measured over tens of meters at Uva (fore-reef rugosity index = 1.51), with centimeter-scale rugosity estimated for individual *Pocillopora* colonies across the Mexican Pacific (rugosity index = 2.95–3.75) showed that framework-scale rugosity is much lower than colony-level rugosity as a result of the tightly-packed structure of *Pocillopora* framework^14,15^. Indeed, the dense *Pocillopora* framework at our sites created an almost-flat surface, which made it particularly challenging to accurately measure rugosity using traditional chain methodologies. The rugosity of the framework would be drastically overestimated if we were to use estimates of colonly-level rugosity. Furthermore, branching colonies actively calcify at their branch-tips, unlike massive species, which actively calcify across their entire surfaces^16^. Because of these complications, and because rugosity was not measured *in situ* at our sites, that variable was not incorporated into our carbonate budget model.

**Infaunal Bioerosion**

The carbonate budget developed by Eakin^1^ for Uva Reef (1988–1994) indicated that cryptofaunal bioerosion was the main bioerosive pressure on the reef at that time (Table S2). Infaunal bioerosion at Uva accounted for >50% of total bioerosion in every reef zone. Eakin used the bioerosion rates estimated by Glynn^17^ for the infaunal bioerosion of *Pocillopora* skeletons, which are the same rates we incorporated into our model. Therefore, different estimates of infaunal bioerosion rates between Eakin’s model and ours are caused by differences in benthic cover.

**Sea-Urchin Densities and Bioerosion**

At each site, six 25 x 1 m video belt transects were deployed haphazardly by SCUBA divers and captured with a GoPro camera, which were pointed down 1 m from the reef surface as they travelled along the transect at a constant speed. The total abundance of *Diadema mexicanum*, the only species that was present, was estimated for each video transect by visually counting each individual seen within the transect area. Since sea urchins are predominantly nocturnal, our density estimates are likely underestimates of the actual population at each site (Fig. S1; ref. ^18^). Sea-urchin bioerosion estimates for each site were then calculated using the rates reported by Glynn^17^ for live, dead, and algal-dominated *Pocillopora* framework and multiplied by the sea-urchin densities from the video transects. Our sea-urchin bioerosion estimates are lower than those estimated by Eakin^1^ (Table S2), which is a result of the difference in sea-urchin densities (Fig. S2). The high sea-urchin densities reported by Eakin^1^ are a consequence of the 1982–1983 El Niño event, which caused extensive coral mortality and an increase in algal cover. This increase in resource availability led to a spike in sea-urchin densities that persisted through the 1980s and the early 1990s. In contrast, when our density estimates are compared with pre-1983 and more recent surveys, they fall within the range of these values.


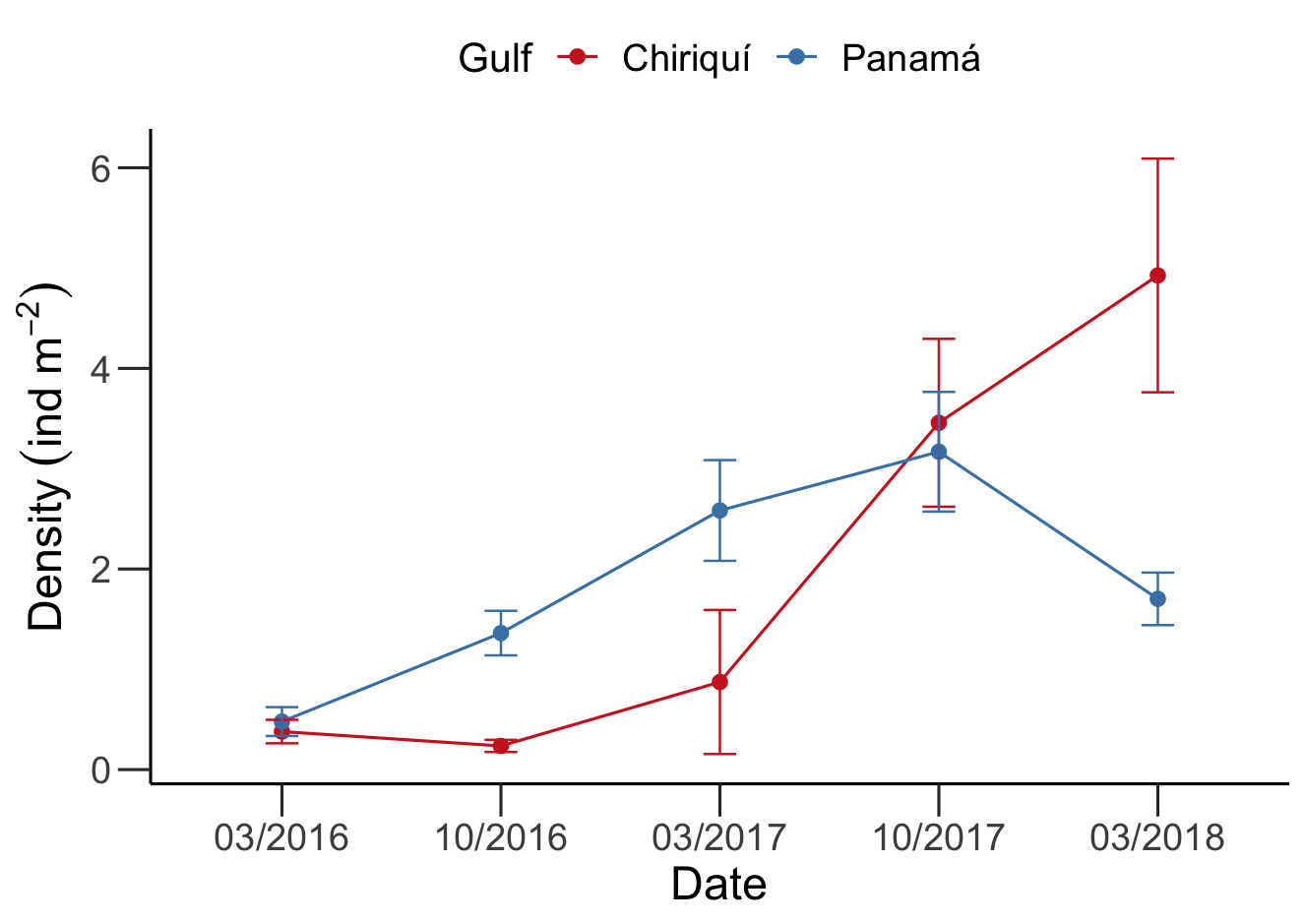


**Figure S1:** Mean (± standard error) density of *D. mexicanum* (ind. m^-2^) for each gulf across the five surveys from 2016–2018.

A regional assessment of sea-urchin populations was recently conducted across the eastern tropical Pacific (ETP)^19^. According to the survey, the average urchin density for the ETP was 0.5 ind m^-2^, and the average bioerosive pressure from sea urchins was 0.16 kg CaCO_3_ m^-2^ yr^-1^ (ref.^19^). During 2009–2010, the average sea-urchin density was 0.7 ind m^-2^ (±0.35) for the Gulf of Panamá, and 0.3 ind m^-2^ (±0.43) for the Gulf of Chiriquí^19^. We estimated the average sea-urchin density for the Gulf of Chiriquí to be 2 ind m^-2^ (±1.64), and 1.9 (±0.86) for the Gulf of Panamá for the period 2016–2018. These values are higher than the estimates from Alvarado et al.^19^ and fall within the range of pre-1983 estimates (2.7 ind m^-2^ ±0.65; Fig. S2).


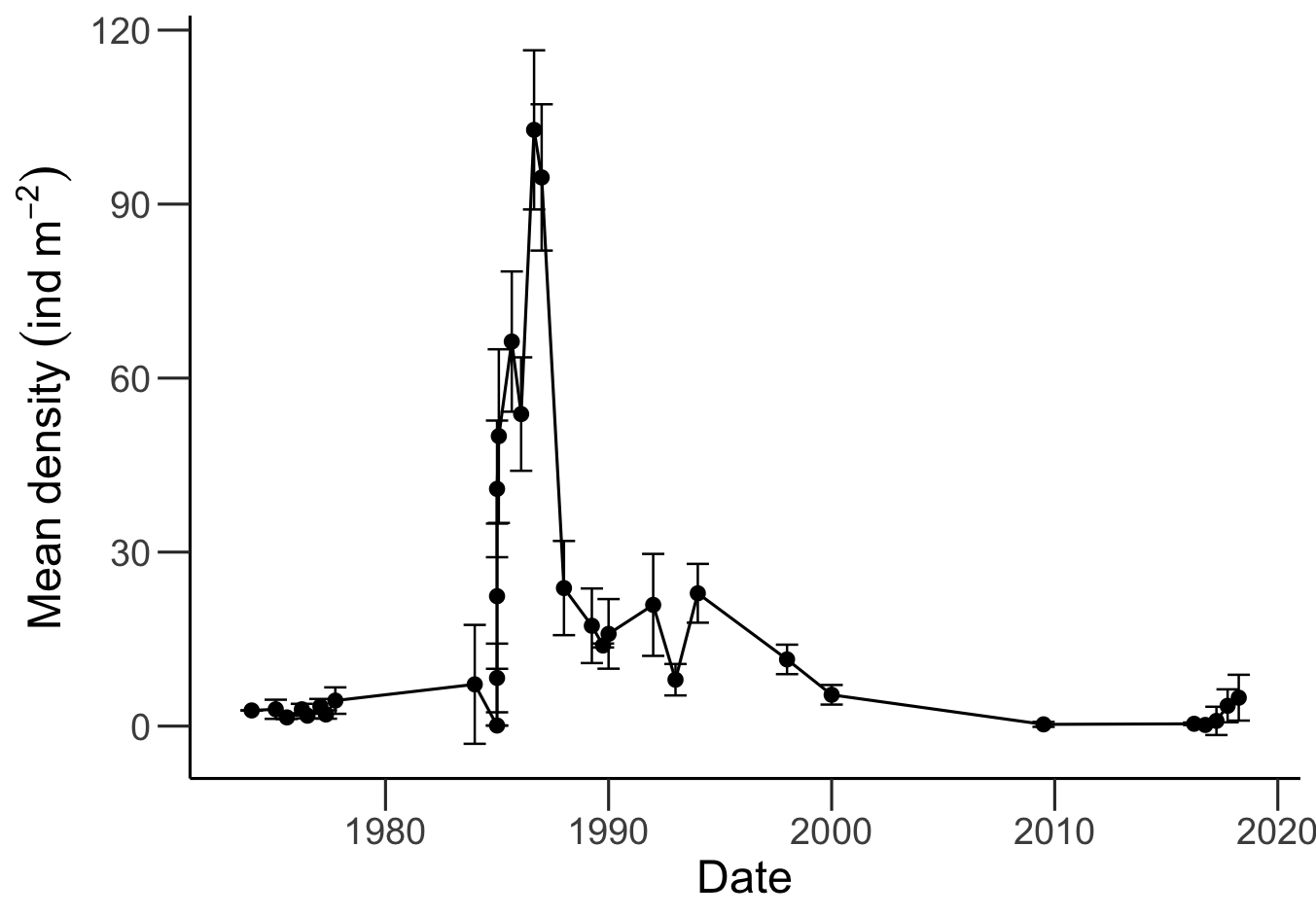


**Figure S2:** Historical estimates *of* *Diadema mexicaum* densities (mean ±95% CI) for the Gulf of Chiriquí. Data were compiled from Glynn^17^, Eakin^14^, Alvarado et al.^19^, and this study to visualize the historical trends in urchin densities at this site.

**Parrotfish Grazing**

The parrotfish species at our sites were *Scarus ghobban* and *S. rubroviolaceus*, the dominant herbivorous fishes on reefs of the ETP^20^. Both of these species scrape the coral substrate while foraging, which is a much less destructive process than excavating. Furthermore, *S. ghobban* does not consistently produce scars while foraging^21^. In other regions, large, excavating parrotfish are the main contributors to the majority of parrotfish-driven bioerosion^22,23^. In the Mexican Caribbean, for example, a decrease in bioerosion rates was driven primarily by a decrease in abundance and a shift towards smaller size classes of the excavating species *Sparisoma viride*. Parrotfish bioerosion decreased from 1.7 kg CaCO_3_ m^-2^ yr^-1^ in 2004 to 0.7 kg CaCO_3_ m^-2^ yr^-1^ in 2018^23^. In the Indian Ocean, large excavators accounted for >60% of total parrotfish bioerosion, with average parrotfish bioerosion being 3.6 kg CaCO_3_ m^-2^ yr^-1^ for the Chagos Archipelago and 3.1 kg CaCO_3_ m^-2^ yr^-1^ for the Maldives^22^. Parrotfish bioerosion estimates of 1.3–1.6 kg CaCO_3_ m^-2^ yr^-1^ have been recorded at the shallow the *Pocillopora* reefs of Gorgona Island, Colombia^20^; however, this was the result of a parrotfish density of 1544 ind. ha^-1^. The parrotfish density at Gorgona Island is almost four times greater than that of the Gulf of Chiriquí, and 50% greater than that of the Gulf of Panamá (Table S7).

**Table S7:** Comparison of parrotfish densities and bioerosion rates estimated for different localities within the eastern Pacific.

| **Site** | **Parrotfish density (ind. ha^-1^)** | **Bioerosion (kg CaCO_3_ m^-2^ yr^-1^)** | **Reference** |
| --- | --- | --- | --- |
| Gulf of Chiriquí | 422 | 0.20 | This study |
| Gulf of Panamá | 970 | 0.46 | This study |
| Gulf of Panamá (upwelling) | 970 | 0.22 | This study |
| Gorgona Island | 1544 | 1.59 | ^20^ |

Although our density estimates for the parrotfish assemblage are lower than the ones reported for the Gorgona Island, our population-density estimates for *S. rubroviolaceous* fall within the range of estimates reported for Uva Island reef between 1980 and 2010^24^ (Fig. S3). On the other hand, our estimates for *S. ghobban* are higher than historical ones^24^ (Fig. S4). Almost all of the individuals recorded at our sites were juveniles, and although juveniles tend to have higher bite rates than adults, a smaller proportion of their bites are significant enough to scar the substrate. The absence of large excavating parrotfish species at our sites explains why parrotfish bioerosion is not the dominant bioeroding agent.


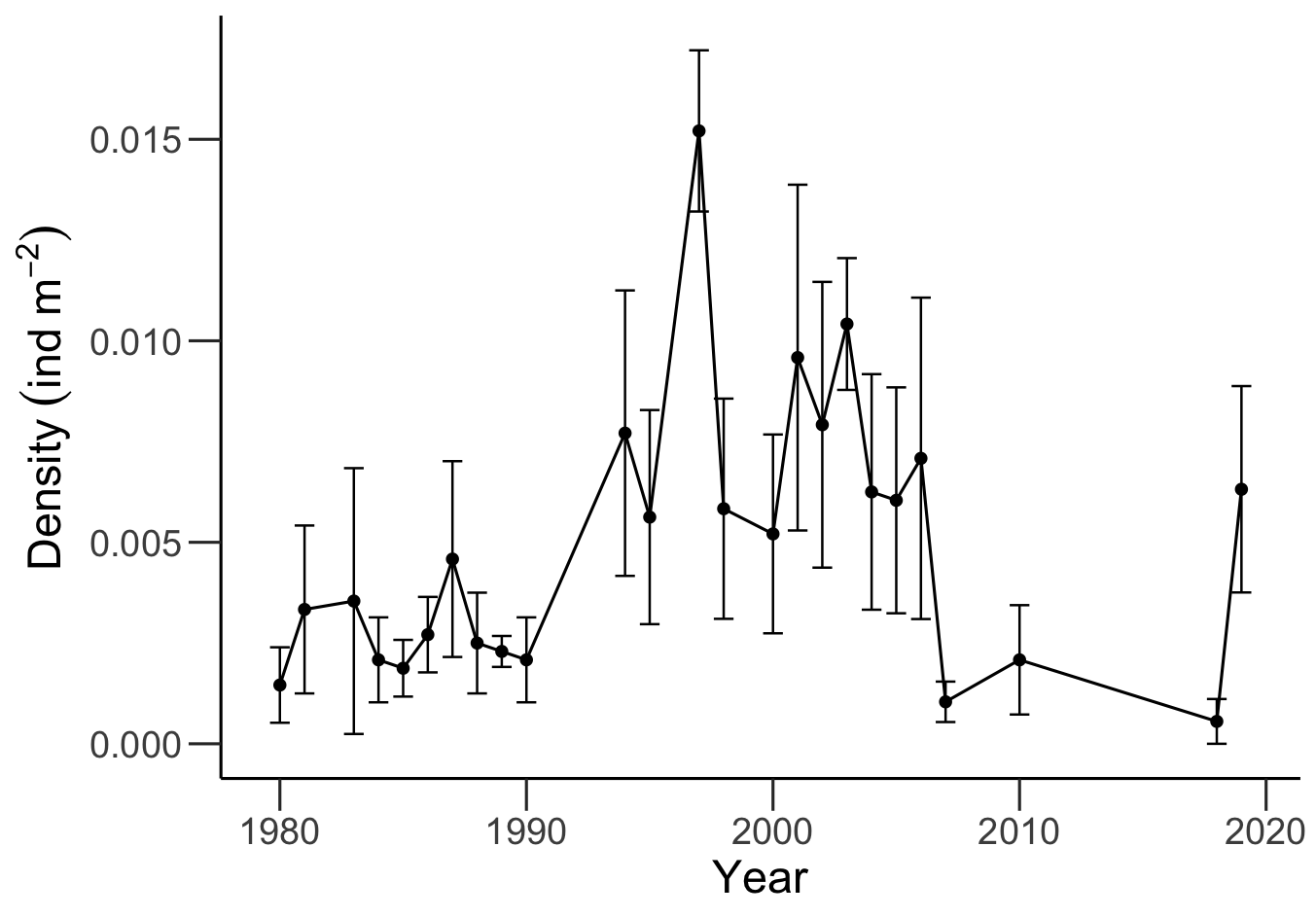


**Figure S3:** Historical (1980–2010) and recent (2018–2019) population-density estimates (ind m^-2^; mean ±95% CI) for *Scarus rubroviolaceous* at Uva Island reef, Gulf of Chiriquí. Historical data were retrieved and modified from Glynn et al.^24^.


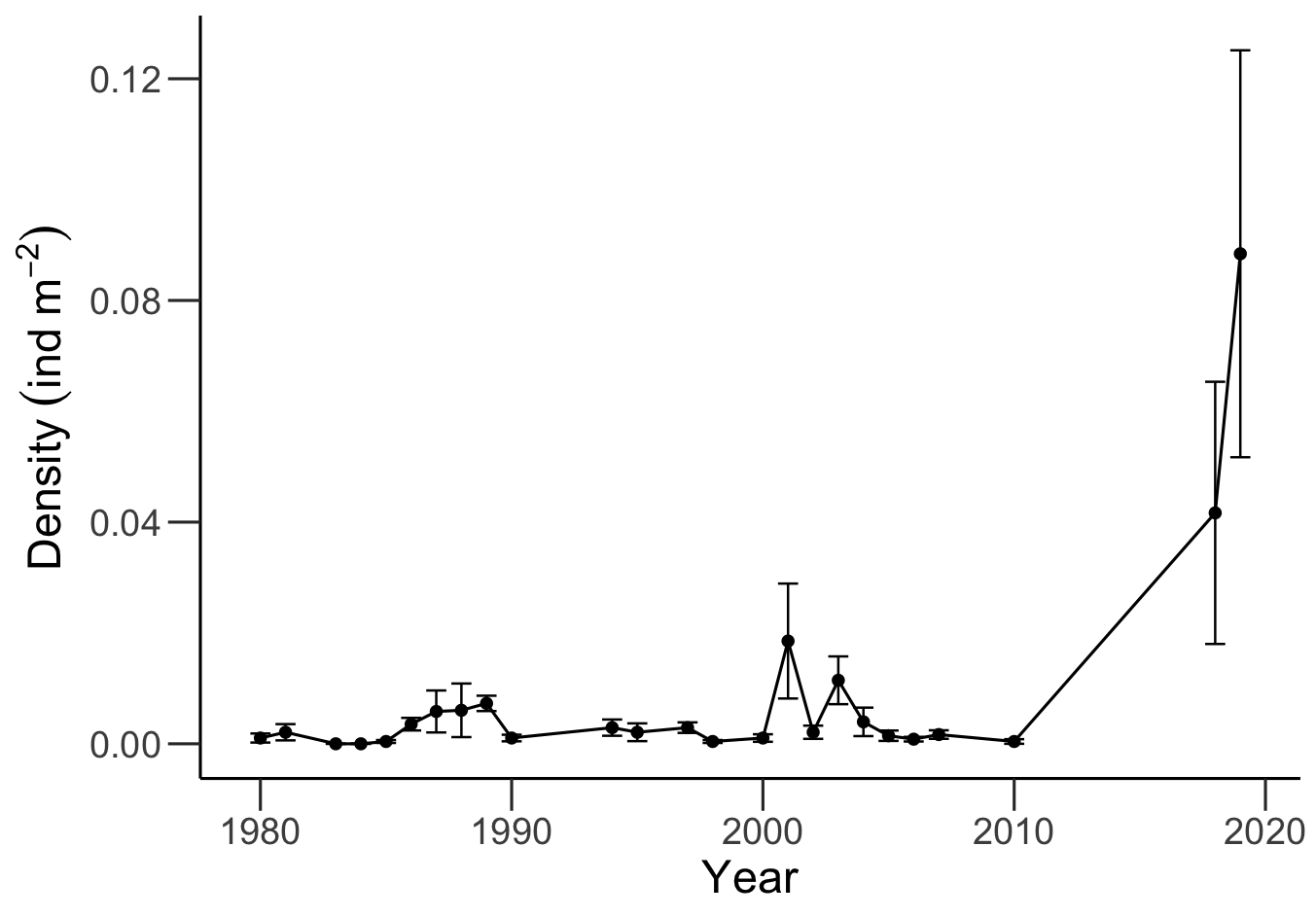


**Figure S4:** Historical (1980–2010) and recent (2018–2019) population-density estimates (ind m^-2^; mean ±95% CI) for *Scarus ghobban* at Uva Island reef, Gulf of Chiriquí. Historical data were retrieved and modified from Glynn et al.^24^.

**Corallivory by *Arothron meleagris***

Multiple studies have assessed the population density and bioerosion rates of the pufferfish *Arothron meleagris* (Tetraodontidae) to determine its contribution to carbonate budgets^25–27^. The population density of *A. meleagris* varies across regions of the eastern Pacific from 0.3 ind ha^-1^ on Contadora Island, Gulf of Chiriquí to 231 ind ha^-1^ recorded at Azufrada Reef, Colombia^27,28^. The average population density of *A. meleagris* across the eastern Pacific is 46.5 ind ha^-1^ (Table S8). In 2018, we estimated an average population density of 0 and 33 ind. ha^-1^ for the Gulf of Panamá and the Gulf of Chiriquí, respectively. In 2019, we estimated an average population density of 0 and 23 ind ha^-1^ for the Gulf of Panamá and the Gulf of Chiriquí, respectively. When compared with historical data^24^, our population-density estimates for *A. meleagris* fall within the range of historical densities for Pacific Panamá (Fig. S5).

**Table S8:** Compilation of studies that have assessed the population density of *Arothron meleagris* on eastern Pacific reefs.

| **Country** | **Locality** | **Site** | **Date** | **Density (ind/ha)** | **Habitat** | **Reference** |
| --- | --- | --- | --- | --- | --- | --- |
| Costa Rica | Caño Island | Caño Island | 1985 | 5 | *Porites* reef | ^28^ |
| Costa Rica | Caño Island | Caño Island | 1986/87 | 11 | *Porites* reef | ^28^ |
| Costa Rica | Caño Island | Caño Island | 1987 | 8 | *Porites* reef | ^28^ |
| Panama | Gulf of Chiriqui | Uva Island | 1981 | 50 | *Pocillopora* reef | ^28^ |
| Panama | Gulf of Chiriqui | Uva Island | 1981/84 | 55 | *Pocillopora* reef | ^29^ |
| Panama | Gulf of Chiriqui | Uva Island | 1986/89 | 55 | *Pocillopora* reef | ^28^ |
| Panama | Gulf of Chiriqui | Secas Island | 1986/88 | 27 | *Pocillopora* reef | ^28^ |
| Panama | Gulf of Panama | Señora Islet | 1971 | 40 | *Pocillopora* reef | ^25^ |
| Panama | Gulf of Panama | Señora Islet | 1987 | 0 | *Pocillopora* reef | ^28^ |
| Panama | Gulf of Panama | Saboga | 1987 | 0 | *Pocillopora* reef | ^28^ |
| Panama | Gulf of Panama | Contadora | 1987 | 0.3 | *Pocillopora* reef | ^28^ |
| Colombia | Gorgona Island | Azufrada reef | 1979 | 12 | *Pocillopora* reef | ^30^ |
| Colombia | Gorgona Island | Azufrada Reef | 1987/88 | 34 | *Pocillopora* reef | ^28^ |
| Colombia | Gorgona Island | Azufrada Reef | 1989 | 25.8 | *Pocillopora* reef | ^31^ |
| Colombia | Gorgona Island | Azufrada Reef | 1993 | 80 | *Pocillopora* reef | ^32^ |
| Colombia | Gorgona Island | Azufrada Reef | 2012 | 231 | Reef crest | ^27^ |
| Colombia | Gorgona Island | Azufrada Reef | 2012 | 192 | Reef flat | ^27^ |
| Colombia | Gorgona Island | Playa Blanca | 1989 | 21.9 | *Pocillopora* reef | ^31^ |
| Mexico | Gulf of California | Cabo Pulmo | 1991/92 | 39 | *Pocillopora* reef | ^26^ |
| Mexico | Tenacatita Bay | Playa Mora | 2002/04 | 43.7 | *Pocillopora* reef | ^33^ |

For the bioerosion rates of *Arothron* *meleagris*, we used the estimates from Palacios et al.^27^. They estimated bioerosion by recording bite-rates and feeding activity in the wild, which yielded estimates that were eight times higher than those from previous studies in aquaria^25,26^.

**
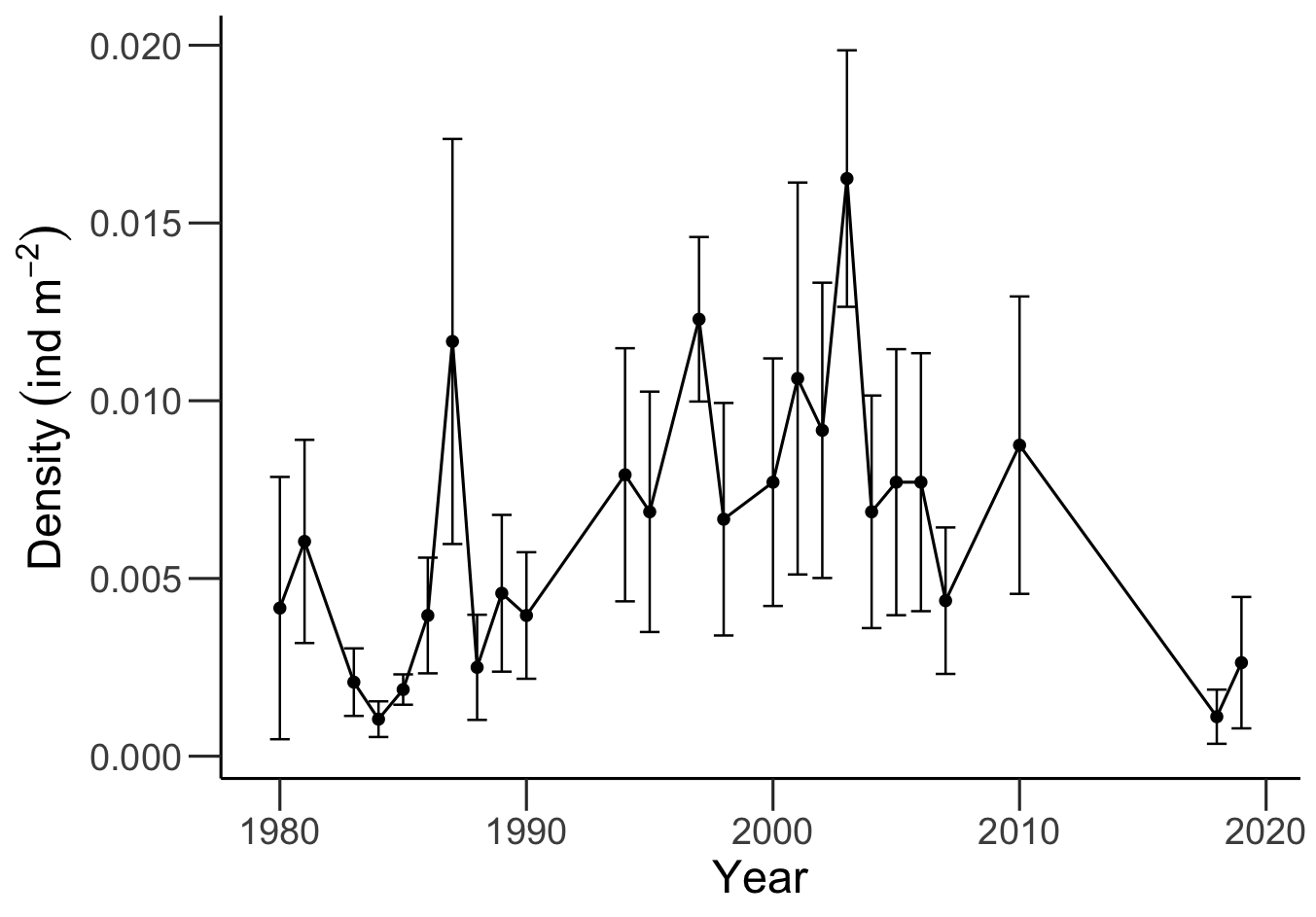
**

**Figure S5:** Historical (1980–2010) and recent (2018–2019) population-density estimates (ind m^-2^; mean ±95% CI) for *Arothron meleagris* at Uva Island reef, Gulf of Chiriquí. Historical data were retrieved and modified from Glynn et al.^24^.

**Sedimentation rates**

Sediments that fill the void spaces of the reef framework provide a significant contribution to reef accretion^34^. To estimate the amount of sediments that were incorporated into the reef framework, we used cores that were previously taken at the same reefs we surveyed to estimate the proportion of the reef framework that was composed of sediment. The relationship between the linear depth of the core samples and the radiocarbon ages, calibrated using the Marine 20 calibration curve^35^, was used to get an estimate of sediment accretion for each gulf in mm yr^-1^. The average sediment-accretion rates for the GoP and the GoC were 0.4 mm yr^-1^ and 0.3 mm yr^-1^, respectively. Since we are using bulk sediment volumes, these estimates include the influence of autochthonous and allochthonous sediments. If we assume that the average density of the sedimentary matrix is 1.1 g cm^-3^, based on estimates from Caribbean reef cores^13^, we get rates of sediment production of 0.4 and 0.3 kg CaCO_3_ m^-2^ yr^-1^ for the GoP and GoC, respectively. These rates of sediment production closely resemble those estimated by Hubbard^34^ for Caribbean reefs.

**References**

1. Eakin, C. M. Where have all the carbonates gone? A model comparison of calcium carbonate budgets before and after the 1982-1983 El Niño at Uva Island in the eastern Pacific. *Coral Reefs* **15**, 109–119. <https://doi.org/10.1007/BF01771900> (1996).

2. Perry, C. T. *et al.* Loss of coral reef growth capacity to track future increases in sea level. *Nature* **558**, 396–400. <https://doi.org/10.1038/s41586-018-0194-z> (2018).

3. van Woesik, R. & William Cacciapaglia, C. Keeping up with sea-level rise: Carbonate production rates in Palau and yap, western pacific ocean. *PLoS One* **13**, e0197077. <https://doi.org/10.1371/journal.pone.0197077> (2018).

4. Manzello, D. P. Coral growth with thermal stress and ocean acidification: Lessons from the eastern tropical Pacific. *Coral Reefs* **29**, 749–758. <https://doi.org/10.1007/s00338-010-0623-4> (2010).

5. Wellington, G. M. & Glynn, P. W. Environmental influences on skeletal banding in eastern Pacific (Panama) corals. *Coral Reefs* **1**, 215–222. <https://doi.org/10.1007/BF00304418> (1983).

6. Tortolero-Langarica, J. de J. A., Rodríguez-Troncoso, A. P., Cupul-Magaña, A. L. & Carricart-Ganivet, J. P. Calcification and growth rate recovery of the reef-building Pocillopora species in the northeast tropical Pacific following an ENSO disturbance. *PeerJ* **2017**, e3191. <https://doi.org/10.7717/peerj.3191> (2017).

7. Medellín-Maldonado, F. *et al.* Calcification of the main reef-building coral species on the Pacific coast of southern Mexico. *Cienc. Mar.* **42**, 209–225. <https://doi.org/10.7773/cm.v42i3.2650> (2016).

8. Cabral-Tena, R. A. *et al.* Different calcification rates in males and females of the coral Porites panamensis in the Gulf of California. *Mar. Ecol. Prog. Ser.* **476**, 1–8. <https://doi.org/10.3354/meps10269> (2013).

9. Norzagaray-López, C. O. *et al.* Low calcification rates and calcium carbonate production in Porites panamensis at its northernmost geographic distribution. *Mar. Ecol.* **36**, 1244–1255. <https://doi.org/10.1111/maec.12227> (2015).

10. Toth, L. T. *et al.* ENSO drove 2500-year collapse of Eastern Pacific coral reefs. *Science* **336**, 81–84. <https://doi.org/10.1016/j.gloplacha.2021.103479> (2012).

11. Hill, J. & Wilkinson, C. Methods for ecological monitoring of coral reefs. *Australian Institute of Marine Science, Townsville* (2004) doi:10.1017/CBO9781107415324.004.

12. Perry, C. T., Lange, I. D. & Januchowski-Hartley, F. A. ReefBudget Indo Pacific: online resource and methodology. *http://geography.exeter.ac.uk/reefbudget/* (2018).

13. Whitcher, E. M. Reef-building threatened by ecosystem decline: A case study from Buck Island, U.S. Virgin Islands. (Florida Institute of Technology, 2017).

14. Eakin, C. M. A tale of two ENSO events: Carbonate budgets and the influence of two warming disturbances and intervening variability, Uva Island, Panama. *Bull. Mar. Sci.* **69**, 171–186. (2001).

15. Cabral-Tena, R. A. *et al.* Functional potential of coral assemblages along a typical Eastern Tropical Pacific reef tract. *Ecol. Indic.* **119**,106795. <https://doi.org/10.1016/j.ecolind.2020.106795> (2020).

16. González-Barrios, F. J. & Álvarez-Filip, L. A framework for measuring coral species-specific contribution to reef functioning in the Caribbean. *Ecol. Indic.* **95**, 877–886. <https://doi.org/10.1016/j.ecolind.2018.08.038> (2018).

17. Glynn, P. W. El Niño warming, coral mortality and reef framework destruction by echinoid bioerosion in the eastern Pacific. *Galaxea* **7**, 129–160 (1988).

18. Mudge, L., Alves, C., Figueroa-Zavala, B. & Bruno, J. Assessment of Elkhorn Coral Populations and Associated Herbivores in Akumal, Mexico. *Front. Mar. Sci.* **6**, 683. <https://doi.org/10.3389/fmars.2019.00683> (2019).

19. Alvarado, J. J., Cortés, J., Guzman, H. & Reyes-Bonilla, H. Density, size, and biomass of Diadema mexicanum (Echinoidea) in Eastern Tropical Pacific coral reefs. *Aquat. Biol.* **24**, 151–161. <https://doi.org/10.3354/ab00645> (2016).

20. Alvarado, J. J., Grassian, B., Cantera-Kintz, J. R., Carballo, J. L. & Londoño-Cruz, E. Coral Reef Bioerosion in the Eastern Tropical Pacific. In Coral reefs of the eastern tropical Pacific (eds Glynn, P. W., Manzello, D. P., Enochs, I. C.) 369–403 (Springer, 2017).

21. Bellwood, D. R. & Choat, J. H. A functional analysis of grazing in parrotfishes (family Scaridae): the ecological implications. *Environ. Biol. Fishes* **28**, 189–214. (1990).

22. Lange, I. D. *et al.* Site-level variation in parrotfish grazing and bioerosion as a function of species-specific feeding metrics. *Diversity (Basel)* **12**, 379. <https://doi.org/10.3390/d12100379> (2020).

23. Molina-Hernández, A., González-Barrios, F. J., Perry, C. T. & Álvarez-Filip, L. Two decades of carbonate budget change on shifted coral reef assemblages: are these reefs being locked into low net budget states? *Proc. R. Soc. B* **287**, 20202305. <https://doi.org/10.1098/rspb.2020.2305> (2020).

24. Glynn, P. W., Enochs, I. C., Afflerbach, J. A., Brandtneris, V. W. & Serafy, J. E. Eastern Pacific reef fish responses to coral recovery following El Niño disturbances. *Mar. Ecol. Prog. Ser.* **495**, 233–247. <https://doi.org/10.3354/meps10594> (2014).

25. Glynn, P. W., Stewart, R. H. & McCosker, J. E. Pacific coral reefs of panamá: Structure, distribution and predators. *Geol. Rundsch.* **61**, 483–519. <https://doi.org/10.1007/BF01896330> (1972).

26. Reyes-Bonilla, H. & Calderon-Aguilera, L. E. Population density, distribution and consumption rates of three corallivores at Cabo Pulmo reef, Gulf of California, Mexico. *Mar. Ecol.* **20**, 347–357. <https://doi.org/10.1046/j.1439-0485.1999.2034080.x> (1999).

27. Palacios, M. M., Muñoz, C. G. & Zapata, F. A. Fish corallivory on a pocilloporid reef and experimental coral responses to predation. *Coral Reefs* **33**, 625–636. <https://doi.org/10.1007/s00338-014-1173-y> (2014).

28. Guzmán, H. & Robertson, D. Population and feeding responses of the corallivorous pufferfish Arothron meleagris to coral mortality in the eastern Pacific. *Mar. Ecol. Prog. Ser.* **55**, 121–131. (1989).

29. Glynn, P. W. Corallivore population sizes and feeding effects following El Nino (1982-1983) associated coral mortality in Panama. *Proceedings of the 5th International Coral Reef Congress* **4**, (1985).

30. Glynn, P. W., von Prahl, H. & Guhl, F. Coral reefs of Gorgona Island, Colombia, with special reference to corallivores and their influence on community structure and reef development. *An. Inst. Inv. Mar.* **12,** 185–214. <https://doi.org/10.25268/BIMC.INVEMAR.1982.12.0.502> (1982).

31. Guzmán, H. M. & López, J. D. Diet of the corallivorous pufferfish Arothron meleagris (Pisces: Tetraodontidae) at Gorgona Island, Colombia. *Rev. Biol. Trop.* **39**, 203–206. (1991).

32. Zapata, F.A.; Morales, Y. A. Spatial and temporal patterns of fish diversity in a coral reef at Gorgona Island, Colombia. in *8th International Coral Reef Symposium* (1997).

33. Galván-Villa, C. M., López-Uriarte, E. & Arreola-Robles, J. L. Diversidad, estructura y variación temporal del ensamble de peces asociados al arrecife coralino de Playa Mora, Bahía de Tenacatita, México. *Hidrobiologica* **21**, 135–146. (2011).

34. Hubbard, D. K. Reefs as Dynamic Systems. In *Life and Death of Coral Reefs* (ed Birkeland, C.) 68–95 (Chapman & Hall, 1997)

35. Heaton, T. J. *et al.* Marine20 - The Marine Radiocarbon Age Calibration Curve (0-55,000 cal BP). *Radiocarbon* **62**, 779–820. <https://doi.org/10.1017/RDC.2020.68> (2020).Eakin, C. M. Where have all the carbonates gone? A model comparison of calcium carbonate budgets before and after the 1982-1983 El Niño at Uva Island in the eastern Pacific. *Coral Reefs* **15**, (1996).
